# Noninvasive prenatal testing for β-thalassemia by targeted nanopore sequencing combined with relative haplotype dosage (RHDO): a feasibility study

**DOI:** 10.1038/s41598-021-85128-2

**Published:** 2021-03-11

**Authors:** Fuman Jiang, Weiqiang Liu, Longmei Zhang, Yulai Guo, Min Chen, Xiaojing Zeng, Yang Wang, Yufan Li, JiaJia Xian, BoLe Du, Yuhuan Xie, Shuming Ouyang, Sheng Li, Yinghong Yang, Chunsheng Zhang, Fei Luo, Xiaofang Sun

**Affiliations:** 1 School of Automation Science & Engineering, South China University of Technology, Guangzhou, 510641 China; 2 Department of Fetal Medicine and Prenatal Diagnosis, Key Laboratory for Major Obstetric Diseases of Guangdong Province, Key Laboratory of Reproduction and Genetics of Guangdong Higher Education Institutes, The Third Affiliated Hospital of Guangzhou Medical University, 63 Duobao Rd., Guangzhou, 510150 China; 3 Shenzhen Jingke Gene Technology Co. Ltd, Shenzhen, 518052 China; 4 Guangzhou Jingke Medical Laboratory, Guangzhou, 510320 China

**Keywords:** Biological techniques, Genetics, Molecular biology, Diseases, Molecular medicine

## Abstract

Noninvasive prenatal testing (NIPT) for single gene disorders remains challenging. One approach that allows for accurate detection of the slight increase of the maternally inherited allele is the relative haplotype dosage (RHDO) analysis, which requires the construction of parental haplotypes. Recently, the nanopore sequencing technologies have become available and may be an ideal tool for direct construction of haplotypes. Here, we explored the feasibility of combining nanopore sequencing with the RHDO analysis in NIPT of β-thalassemia. Thirteen families at risk for β-thalassemia were recruited. Targeted region of parental genomic DNA was amplified by long-range PCR of 10 kb and 20 kb amplicons. Parental haplotypes were constructed using nanopore sequencing and next generation sequencing data. Fetal inheritance of parental haplotypes was classified by the RHDO analysis using data from maternal plasma DNA sequencing. Haplotype phasing was achieved in 12 families using data from 10 kb library. While data from the 20 kb library gave a better performance that haplotype phasing was achieved in all 13 families. Fetal status was correctly classified in 12 out of 13 families. Thus, targeted nanopore sequencing combined with the RHDO analysis is feasible to NIPT for β-thalassemia.

## Introduction

The application of noninvasive prenatal testing (NIPT) for single gene disorders (SGDs) remains challenging. It was first applied to detect the sequences inherited from the father or to exclude the paternal mutation, such as sex linked diseases^1^, rhesus D blood group testing^2^, congenital adrenal hyperplasia^3^ and so on. Recently, noninvasive prenatal single-gene screening tests are commercially available. For example, Vistara (Natera Inc., San Carlos/CA, USA) NIPT screens for several conditions and identifies risk for SGDs in a spectrum of genes. In most cases, the test screens for new mutations that inherited in an autosomal or X-linked dominant fashion, such as FGFR3-related Achondroplasia, MECP2-related Rett syndrome. which means that if the mutation is present, the child will be affected by the condition and experience related symptoms.

For SGDs of maternal origin, it is a challenge to accurately detect and quantify the slight increase of the maternally inherited allele contributed by the fetal DNA. To solve the problem, different approaches such as relative mutation dosage (RMD)^4^ and relative haplotype dosage (RHDO)^5^ have been developed. RMD is based on analyzing balance or imbalance of wild-type and mutant alleles in maternal plasma using digital PCR or next generation sequencing (NGS), enabling the detection of β-thalassemia^4,6^, sickle cell anemia^7^ and so on. Instead of measuring the allelic alterations related only to one specific mutation site, RHDO assesses the balance of haplotypes in maternal plasma. The haplotypes are constructed using heterozygous single nucleotide polymorphism (SNP) alleles linked in and around the gene of interest to either the mutated or wild-type allele. Inheritance of alleles within the same haplotype can be inferred simultaneously. Compared with RMD approach, RHDO analyzes a range of SNP allele counts within a haplotype, which undoubtedly makes the statistical test more robust^5^. This strategy was successfully applied in several types of SGD studies, such as spinal muscular atrophy^8^, Duchenne and Becker muscular dystrophies^9^, β-thalassemia^5,10^, congenital adrenal hyperplasia^11,12^, Ellis-van Creveld syndrome, hemophilia and Hunter syndrome^13^ and so on.

Prior construction of parental haplotypes is required for RHDO analysis. The conventional haplotyping procedure needs a proband sample^14,15^. However, the DNA sample for proband is sometimes not available. More recently, platforms that provide long reads were commercially available^16–18^. The Oxford Nanopore sequencing technology promotes some of the most complex regions of the human genome to be haplotype phased, such as HLA^19^, CYP2D6^19^, MHC^20^ and Y chromosome^21^. Each long read spans multiple heterozygous SNPs, making the parental haplotype analysis that was previously intractable more accurate and less complex. In this study, we explored the feasibility of applying nanopore sequencing technology combined with the RHDO analysis in NIPT for β-thalassemia, an autosomal recessive blood disease, enabling to build an accurate, cost-effective and convenient method for prenatal testing for SGDs.

## Results

### Clinical participants

The gestational age of the 13 pregnant women varied from 11 to 28 weeks (Table 1). A total of six types of mutations were observed in the 13 families, including *HBB*:c.316-197C > T, *HBB*:c.126_129delCTTT, *HBB*:c.217dup, *HBB*:c.52A > T, *HBB*:c.-78 A > G, *HBB*:c.-79 A > G. In the family 09, 10, 11, 12 and 13, the husband and the wife carried the same mutation.

**Table 1:** Summary of family information and NIPT results included in this study.

Family	Mate-rnal age (years)	Gestatio-nal age	Maternal genotype	Paternal genotype	Fetal fracti-on (%)	CVS	NIPT results	
							10 kb library	20 kb library
F01	31	21w	*HBB*:c.316-197C > T	*HBB*:c.126_129delCTTT	12	Affected	Affected	Affected
F02	28	28w	*HBB*:c.52A > T	*HBB*:c.316-197C > T	20	Affected	Affected	Affected
F03	-	21w	*HBB*:c.316-197C > T	*HBB*:c.126_129delCTTT	17	Affected	Affected	Affected
F04	27	18w	*HBB*:c.-78 A > G	*HBB*:c.52A > T	13	Affected	Affected	Affected
F05	-	18w	*HBB*:c.126_129delCTTT	*HBB*:c.-78 A > G	18	Carrier	WT/ *HBB*:c.-78 A > G	WT/ *HBB*:c.-78 A > G
F06	27	12w	*HBB*:c.52A > T	*HBB*:c.316-197C > T	20	Affected	Affected	Affected
F07	24	19w + 2d	*HBB*:c.126_129delCTTT	*HBB*:c.217dup	14	Carrier	WT/ *HBB*:c.217dup	WT/ *HBB*:c.217dup
F08	30	16w + 6d	*HBB*:c.52A > T	*HBB*:c.-79 A > G	8	Affected	Affected	Affected
F09	30	20w	*HBB*:c.126_129delCTTT	*HBB*:c.126_129delCTTT	12	Carrier	inconclusive	WT/ *HBB*:c.126_129delCTTT
F10	23	17w	*HBB*:c.52A > T	*HBB*:c.52A > T	10	Affected	inconclusive	inconclusive
F11	28	11w	*HBB*:c.316-197C > T	*HBB*:c.316-197C > T	19	Affected	Affected	Affected
F12	35	18w + 4d	*HBB*:c.316-197C > T	*HBB*:c.316-197C > T	9	Normal	WT/WT	WT/WT
F13	39	16w + 2d	*HBB*:c.126_129delCTTT	*HBB*:c.126_129delCTTT	11	Affected	Affected	Affected

### Performance evaluation of MinION sequencing

**Figure 1:**
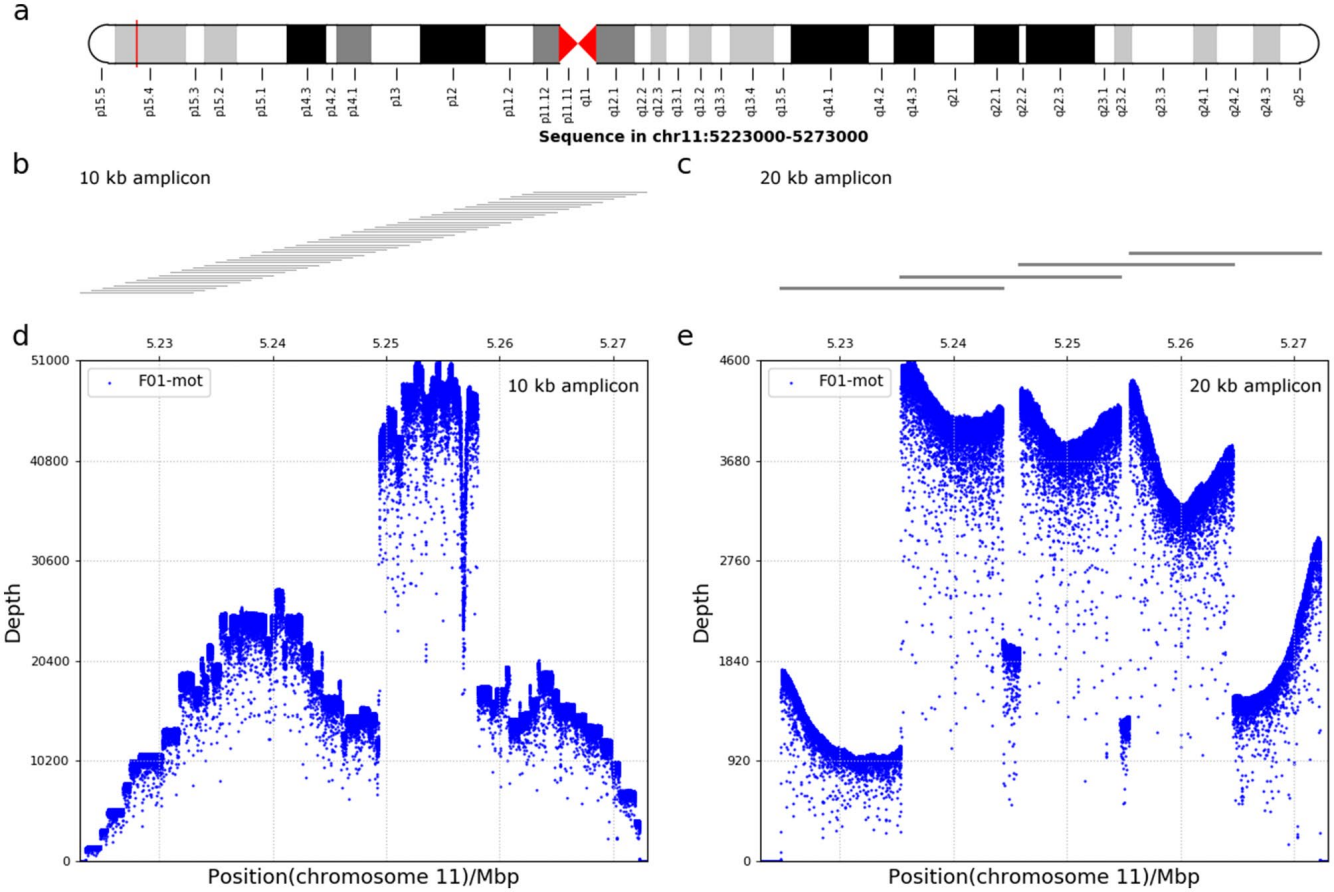
Targeted region of MinION sequencing. (**a**) Diagram of the targeted region locus on chromosome 11 containing the *HBB* gene selected for this study was indicated by the red vertical line. (b and c) Long-range PCR strategy used for haplotyping. PCR amplicons of 10 kb and 20 kb libraries was indicated in (**b**,**c**) respectively. 10 kb or 20 kb PCR amplicons were designed overlapped and each horizontal line represents an amplicon. (**d**,**e**) The depth distribution and the coverage of sequencing data in the whole targeted region. Examples of the depth distribution of targeted region by MinION sequencing for 10 kb and 20 kb libraries was indicated in (**d**,**e**) respectively. The horizontal axis represents the position on chromosome 11. The vertical axis represents sequencing depth.

For 10 kb amplicon library, the raw sequencing data was generated from two flow cells. Six samples of the family 01, 02 and 03 were loaded on one flow cell. A total of 752,000 reads yielding 5.42 Gb were acquired, of which 686,085 reads passed quality filters and barcode-recognizing. The average sequencing depth was ~ 15,000 × , of which 97.61% reads above 500 × (Fig. 1d and Table S1). The rest samples were loaded on the other flow cell, which generated 1,208,126 reads summing to over 9.37 Gb data. Higher multiplex level led to fewer DNA molecules to be recognized and a total of 672,967 reads were obtained for further analysis. The average sequencing depth was ~ 5000 × and the sequencing depth above 500 × was obtained in 95.21% of the analyzed reads. On average for all cases from two flow cells, 99.26% of the reads aligned to the targeted region. The average N50 read length was 9 kb and the N50 data was fairly even among the different samples. Alignment of 39 PCR amplicons yielded a complete coverage of the 50 kb targeted region (Fig. 1b).

For 20 kb library, a total of 398,667 pass reads were obtained for analysis. 98% of the reads were aligned to the targeted region, with the average sequencing depth of 2342 × (Fig. 1e). The sequencing depth above 200 × was obtained in 90.89% of the analyzed reads. The average N50 size was 14 kb, ranging from 7 to 17 kb (Table S2). Alignment of 4 PCR amplicons yielded a complete coverage of the targeted region (Fig. 1c).

### Assembly polishing by NGS data

For 10 kb library, nanopore data revealed 84.8–100% SNPs of NGS data detected in the different samples. Take the mother of the family 01 for example, 107 SNPs were detected in the NGS data. Of these 107 SNPs, 93 SNPs (86.9%) were also detected in the nanopore data, of which 90 SNPs showing good consistency (Fig. S1a). Fourteen SNPs that was identified in the NGS data were not detected in the nanopore data. In 93 shared SNPs, 49 SNPs were heterozygous and the rest were homozygous. The detail of the comparison between nanopore sequencing data and NGS data for every sample was listed in Table S3. For SNPs detected by both platforms, ~ 3% SNPs were inconsistent. We noticed that substitution errors tended to occur at the same nucleotide position across different samples. The most common discrepant variant was at position 5,270,539 where guanine was classified as adenine in the nanopore data, which was observed in 19 samples. The corresponding genotype was heterozygous “AC” in NGS data but homozygous “AA” in nanopore data. The homozygous genotype was confirmed by Sanger sequencing (Fig. S2). The second most common discrepancy was at position 5,248,842 where adenine was classified as guanine in the nanopore data. The genotype call was homozygous “AA” in NGS but heterozygous “AG” in nanopore data. The homozygous genotype was confirmed by Sanger sequencing (Fig. S2).

For 20 kb library, nanopore data revealed 85.0–98.2% SNPs of NGS data showed (Fig. S1a and Table S4) and the most two common discrepant SNPs were the same as those in 10 kb library.

Notably, the pathogenic mutations, including point mutation sites or insertions and deletions (indels), were all correctly recognized by nanopore sequencing in both libraries.

### Long-read based parental haplotype phasing

**Figure 2:**
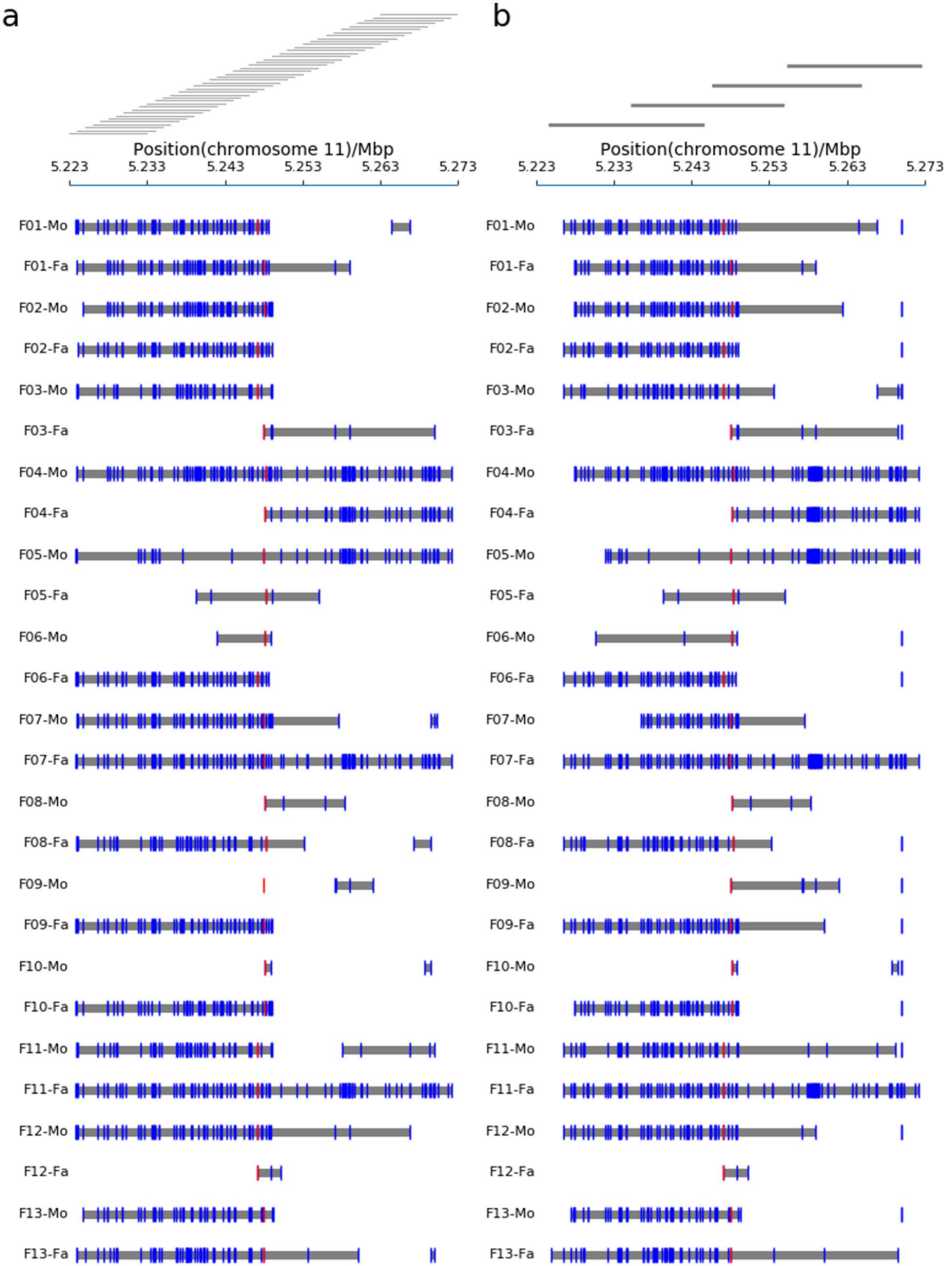
Haplotype phasing results of targeted region for all 13 families using 10 kb (a) and 20 kb (b) libraries, respectively. Haplotype blocks are represented by gray line. Vertical blue lines indicate the SNPs. Vertical red lines indicate the mutation site.

For 10 kb library, haplotypes were successfully constructed in all parental samples except the mother of family 09 (Fig. 2a). The number of heterozygous SNPs in phased haplotypes varied from 2 (F10-Mo) to110 (F04-Mo) per sample. The haplotype blocks ranged from 643 bp to 48 kb in length, with an average length of 26 kb. The maternal haplotype phasing of family 09 was failed to link the mutated or wild type allele to nearby heterozygous SNPs because the nearest heterozygous SNP to the mutation site was located ~ 10 kb downstream.

For 20 kb library, the maternal and paternal haplotypes of 13 families were successfully phased. The average length of haplotype was 27 kb. In the maternal sample of family 09, the pathogenic variant was successfully linked to the nearby heterozygous SNP. And the SNP was served as the anchor to be phased to surrounding SNPs, yielding a ~ 14 kb long haplotype region encompassed 5 SNPs (Fig. 2b). The sequenced data from 20 kb amplicons further confirmed that all haplotypes were correctly phased in the 10 kb amplicons.

### Determination of fetal HBB genotype by RHDO

RHDO analysis was applied to determine the haplotypes inherited by the fetus. The average sequencing depth of maternal plasma DNA was ~ 500 × (range: 317 ~ 895 fold). The fetal fraction ranged from 8 to 20% (Table S5).

The informative SNPs across the whole haplotype were classified into type α and type β (see Methods), and counted separately. For clarity, taken the family 01 as an illustration, in the maternally inherited haplotype analysis of 10 kb library, a total of 3 type α SNPs and 17 type β SNPs were identified (Table S6) from plasma data. For type α SNPs, the reads on Hap 0 were over-represented in 1 SPRT classification. For type β SNPs, the reads on Hap 1 were equal-represented in 6 SPRT classifications. Both analyses indicated that Hap 0 has been inherited by the fetus from the mother. In the paternally inherited haplotype analysis, 11 type α SNPs and 3 type β SNPs were identified. For type α SNPs, the reads on Hap 1 were absent in 9 SPRT classifications. For type β SNPs, The reads on Hap 0 were present in 3 SPRT classifications. Both analyses indicated that Hap 0 has been inherited by the fetus from the father. Thus, we predicted the fetus was affected by β-thalassemia. The same processes were applied to other families (Tables S6 and S7).

Fetus status has been successfully deduced in 12 at-risk pregnancies. The fetus status of family 09 was inconclusive in 10 kb library, because the maternal mutated allele failed to link to the nearby haplotype. In the family 10, the paternally inherited haplotype could be determined. However, the limited heterozygous SNPs nearby the maternal mutation site in the haplotype region hampered the inference of fetus-inherited maternal haplotype in both 10 kb and 20 kb libraries. The logarithmic odds ratios of transmission probability between Hap 0 alleles and Hap 1 alleles were also calculated at each informative SNP site across the whole haplotype region (Fig. S3). Despite some fluctuations, the overall trends were consistent with the RHDO analysis results.

In all cases we could predict that eight fetuses were affected by β-thalassemia, three fetuses were carriers and one fetus was unaffected by β-thalassemia. The predictions were confirmed by invasive prenatal testing, with an accuracy of 100% (Table 1).

## Discussion

In this study, targeted region of parental gDNA containing the *HBB* gene was captured by long-range PCR amplification. Parental haplotypes were phased by combining the nanopore sequencing data and Illumina NGS data. Inheritance of parental haplotypes was classified by the RHDO analysis through the maternal plasma DNA sequencing data. The method had been successfully tested for an early assessment of the fetal β-thalassemia profiles in 12 out of 13 at-risk pregnancies, without the need to include a proband, highlighting the unique advantage of long-read sequencing applied in NIPT for β-thalassemia.

The nanopore sequencing technology is attractive to people who studying NIPT for SGDs because it could produce long reads that were directly used to provide full length parental haplotypes. Recently, two methods combining the RHDO analysis were demonstrated that NIPT for SGDs can be carried out without a proband: the 10 × Genomics technology and targeted locus amplification (TLA) method. Using 10 × Genomics technology followed by NGS^13^, the short DNA molecules derived from long DNA molecules were barcode-tagged, sequenced and assembled into larger contigs based on barcode similarity^18^. However, the method requires specific 10 × Genomics instruments that are too expensive for clinical practice. Alternatively, Vermeulen et al. implemented the TLA method to perform haplotyping^22^. Parental haplotypes were constructed by crosslinking in blood cells, digestion, and proximity ligation primarily to yield intra-chromosomal ligation products. Then the products were selectively amplified by inverse PCR and sequenced. Variants ending up in the same ligation product were assigned to the same allele. This approach was successfully predicted the mutational inheritance status of fetuses in 18 pregnancies, including *CFTR*, *CYP21A2*, and *HBB* genes^23^. However, the TLA method used in haplotype construction requires an additional chromosomal ligation process and is still laborious.

The targeted nanopore sequencing method used in this study is cost-effective. First, parental haplotypes were constructed without a proband. This will decrease the cost of the analysis. Second, targeted enrichment decreases the cost of sequencing the region of interest to sufficient coverage. What is more, compared with other targeted enrichment methods such as hybridization, PCR-based targeted sequencing can provide both targeted enrichment and amplification in a single step, which is relatively cheap. Third, the portable MinION sequencer also has a low cost which offers accessibility to testing in many research and clinical labs. The R9.4 flow cell, approximately $1300, yielded 9 Gb of sequence data with a mean depth of ~ 5000 × in this study, which is much more than adequate for accurate variant calling and phasing. The maximum that can currently be multiplexed on a single flow cell was 96 samples, so the cost would further reduce. Considering the sample preparation and correction with short Illumina reads, we estimated that the overall sequencing cost for materials and reagents was about $400 for a family, making our approach practical for clinical use.

Nanopore sequencing is still incompatible with applications on SNP resolution. The majority of discrepancies in this study were where NGS data were heterozygous but homozygous in nanopore data. We considered the discrepancies were mostly likely to be basecaller errors of nanopore sequencing. Development of dedicated SNP callers for nanopore data will most likely enhance the ability to extract reliable SNPs in the future. Meanwhile, during the preparation of this manuscript, the new chemistry R10 was released into early access and has delivered Q50 in a small genome in internal company experiments. Considering the rapid improvements in this technique, it may not be very long before this sequencing technology potentially produces increased read accuracy.

In this study, long-range PCR of 10 kb amplicons was used to enrich the targeted region first. The 10 kb amplicon library which had an average N50 read length of 9 kb could be effectively used for haplotype assembly of all parental haplotypes except one maternal haplotype. Since the nearby heterozygous SNPs on the same reads are used to phase haplotypes, for the SNPs that are further apart than a read length, whether they are present on the same chromosome is unknown. The optimization of amplicons to 20 kb permitted construction of all 13 parental haplotypes. Long-range PCR was out of necessity since it could amplify the targeted region to generate sufficient material for sequencing at the same time. This is very important, especially for diagnostic applications. The quantity of DNA sample is sometimes limited and a large amount of high molecular weight (HMW) genomic DNA for every sample is required prior to library preparation. Meanwhile, only 4 overlapped PCR amplicons were designed to achieve the haplotypes construction of the same region in 20 kb library, which greatly simplified the library preparation work. Laver T.W. et al.^24^ have reported the formation of chimeric molecules in the long-range PCR amplification step which would render it impossible to call the true haplotypes. We did not observe the presence of chimeras in this region. While the MinION sequencing requires at least 1 ug of input HMW DNA for each sample in the current protocol provided by Oxford Nanopore technologies, we believed less DNA starting quantity demand could be achieved by continuously optimized library construction process. As the required starting DNA quantity reduced, PCR-free way could be used instead to generate the MinION library in future studies.

In the family 10, we could not give a robust result that whether the fetus was a carrier or affected by β-thalassemia. Because the majority of SNPs in maternal haplotype of family 10 were homozygous, the small quantity of informative SNPs usable for the RHDO analysis made a statistically significant result not reached. In clinical practice, the NIPT result was inconclusive rather than false prediction, and the couples could choose an invasive test for further validation.

In this study, our data showed that targeted nanopore sequencing-based haplotyping coupled with the RHDO analysis could be successfully used for NIPT for β-thalassemia. Our results are encouraging, as our workflow could produce reliable results without proband contribution and cost-effective. To took full advantage of nanopore sequencing to generate long reads that could be used for reliable SNP phasing, we corrected the nanopore sequencing data by conducting the NGS sequencing of the long-range PCR products. The long-range PCR has two major advantages. First, the PCR products can be used in the nanopore sequencing and NGS sequencing simultaneously. Second, the enrichment of the targeted region was achieved by direct amplification of the selected region. The barcodes used to distinguish different samples can be also added in this process, which could significantly reduce the cost and make it ideal for clinical settings. Therefore, this approach may represent a valuable resource and we can expect to extend it to other SGDs. However, some obstacles need to be overcome in order for clinical use. The evaluation on the feasibility of this approach still need validation by sufficient clinical samples. Moreover, the errors in nanopore sequencing in SNP calling make it still unsuitable for the moment for clinical use. It can be speculated that improvements in base-calling accuracy of nanopore sequencing in future would greatly facilitate the development of NIPT for SGDs in clinical practice.

## Methods

### Study design

Construction of parental haplotypes is needed in RHDO analysis of NIPT for β-thalassemia. The haplotypes are constructed using heterozygous SNP alleles linked in and around the gene of interest to either the mutated or wild-type allele. The distance between the nearest heterozygous SNP and the mutation site is often more than 10 kb. Therefore, it is currently impossible to identify carriers of such haplotypes directly by Sanger sequencing or NGS platforms because they cannot generate reads longer than ~ 1500 bp from a single DNA molecule. Here, we chose the nanopore sequencing to produce long reads. Since the nanopore sequencing is still incompatible with applications on SNP resolution, the SNP results should be verified on the same sample by high-accuracy methods such as Illumina or Sanger sequencing. Sanger sequencing remains the gold standard for molecular diagnosis when a single-gene disorder is suspected and for confirmation of NGS findings. However, Sanger sequencing is not a feasible approach for routine testing as it is expensive and time-consuming. NGS which is a high-throughput sequencing method permits rapid and cost-effective simultaneous screening of large number of individuals. Therefore, we chose NGS to verify the SNPs detected by the nanopore sequencing here.

**Figure 3:**
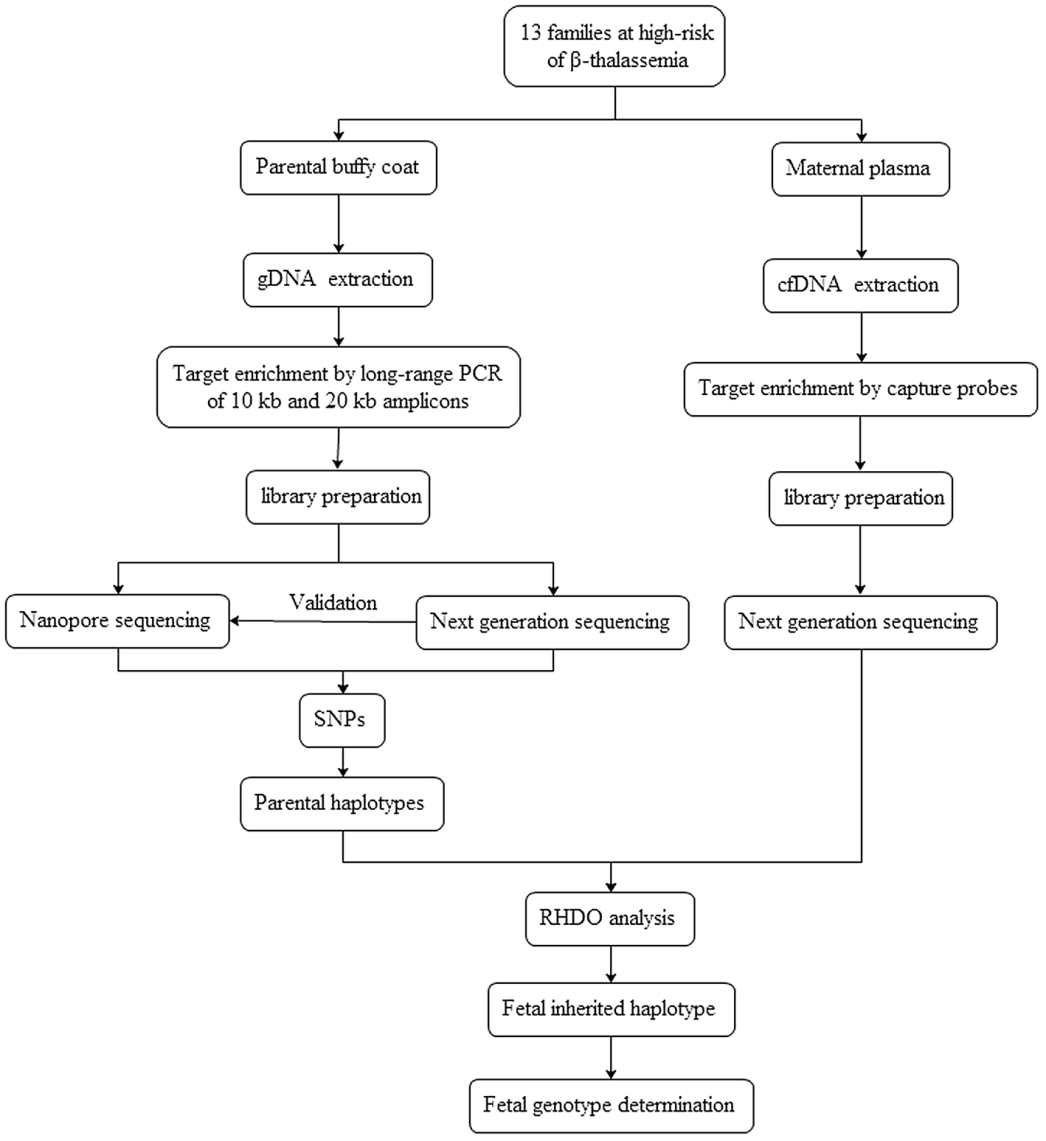
The work flow of this study.

The work flow of this study was shown in Fig. 3. Parental genomic DNA (gDNA) was targeted by long-range PCR of 10 kb and 20 kb amplicons. After library preparation, the products were subjected to the nanopore sequencing and NGS. The SNPs were identified and then parental haplotypes were constructed. Maternal plasma cell-free DNA (cfDNA) was subjected to NGS. Fetal inheritance of parental haplotypes was classified by the RHDO analysis.

### Subjects and samples

Thirteen families at high-risk for β-thalassemia were recruited at the Third Affiliated Hospital of Guangzhou Medical University. Informed consent was obtained from all participants. The study was approved by the ethics committee of the Third Affiliated Hospital of Guangzhou Medical University ([2017] No. 056). All experimental procedures were performed in accordance with relevant guidelines and regulations. Ten mL maternal blood was drawn into Streck tube and 5 mL paternal peripheral blood was drawn into EDTA tube. CfDNA was extracted from maternal plasma with QIAamp Circulating Nucleic Acid Kit (Qiagen) and parental gDNA was extracted from the buffy coat with QIAamp DNA mini kit (Qiagen). Fetal genotype information was obtained through analysis of amniocentesis or chorionic villus samples.

### Nanopore sequencing

The ~ 50 kb targeted region containing the *HBB* gene (chr11: 5,223,000–5,273,000 with reference to GRCh37/hg19, Fig. 1a) was amplified by long-range PCR of 10 kb and 20 kb amplicons, respectively. PCR products were end repaired, A-tailed and sample special-barcode ligated. The fragments were size-selected (10–50 kb) with a BluePinpin (Sage Science). The size distribution of the libraries was analyzed using an Agilent 2100 bioanalyzer and library concentration was quantified by Qubit (Life Technologies). Then the sequencing libraries were prepared employing the Oxford ligation sequencing kit SQK-LSK108 (Oxford Nanopore Technologies) and pooled in equal concentration using native barcoding expansion kit EXP-NBD103 (Oxford Nanopore Technologies) according to recommended procedure. The library was subjected to 48 h reads generated on the MK1B MinION platform using the R9.4 (FLO-MIN106) flow cell (Oxford Nanopore Technologies). Reads were then base-called using MinKNOW software (MinKNOW version 1.15.4, Oxford Nanopore Technologies). All sequenced reads, which were shorter than 2000 bp or had an average quality score lower than 10 were excluded from downstream analysis using Nanofilt (version 2.2.0). Clean reads were aligned on GRCh37 human reference genome with Minimap2 (version 2.11). Variants were called with Nanopolish (version 0.10.1).

### Next generation sequencing

For gDNA sequencing library preparation, PCR products were sheared into ~ 250 bp fragments and converted to library with KAPA Hyper Prep Kit (KAPA Biosystems). Each library was amplified by PCR and then pooled together for sequencing.

For cfDNA sequencing library preparation, the targeted DNA covering 0.69 Mb was enriched by designed biotinylated probes with the NimbleGen SeqCap EZ Choice (Roche) according to the manufacturer’s standard protocol. The cfDNA was end repaired, A-tailed and adaptor ligated with KAPA Hyper Prep Kit (KAPA Biosystems). Then, the 8-bp barcode was added to each sample through PCR and followed by hybridization. Then the captured DNA was amplified by PCR and pooled together for sequencing.

The DNA libraries were subjected to 150-bp paired-end sequencing on NovaSeq platform (Illumina). All sequenced reads, after low quality reads had been filtered, were aligned on GRCh37 human reference genome with BWA-MEM (version 0.7.17-r1188). Duplicated reads were deleted. Variants were called with GATK 3.7 from sequencing data of gDNA. The sequencing depth was determined for each base from sequencing data of cfDNA.

### Direct haplotype phasing

Parental heterozygous SNPs from NGS-validated nanopore sequencing data were used to construct the parental haplotypes. Heterozygous indels detected by NGS were also used to assemble the haplotype. A custom haplotyping script was used to construct haplotypes. This script was designed to allow construction of all possible haplotypes. The most reliable links were selected based on (i) total reads number: more than 10 reads supporting the links; (ii) more than 50% of all supporting reads; (iii) logic: assuming all heterozygous SNPs are bi-allelic, and therefore when one variant is attributed to one haplotype, the other variant can automatically be assigned to the other haplotype. We classified the haplotype contained a deleterious mutation as Hap 0 and the other haplotype without a mutation as Hap 1.

### RHDO analysis

The fetal inherited haplotype was determined by the RHDO analysis as previously described^5^. Briefly, the maternally inherited haplotype was determined by the RHDO analysis carried out at maternally heterozygous but paternally homozygous SNP sites. Each SNP was classified as type α or type β. Allele on maternal Hap 0 which was the same as paternal allele was classified as type α SNP. Allele on maternal Hap 1 which was the same as paternal allele was classified as type β SNPs. Determination whether the Hap 0 or Hap 1 was more likely inherited by the fetus depends on the dosage imbalance of SNPs on Hap 0 and Hap 1. If the fetus inherited Hap 0, type α SNPs would be examined as an overrepresentation. Otherwise, if the fetus inherited Hap 1, alleles on Hap 0 and Hap 1 would be equally represented. Similarly, if the fetus inherited Hap 1, type β SNPs would be overrepresented. While if Hap 0 was transmitted to the fetus, alleles on Hap 0 and Hap 1 would be equally represented. The paternally inherited haplotype was determined in the similar way by using SNPs that were homozygous in the mother and heterozygous in the father. The sequential probability ratio test (SPRT) was used to evaluate if the dosage imbalance was sufficient to reach a statistical conclusion. The fetal fraction was estimated using FetalQuant algorithm^25^.

## Supplementary Information

Supplementary Legends.

Supplementary Figure S1.

Supplementary Figure S2.

Supplementary Figure S3.

Supplementary Tables.

## Data Availability

All data generated or analysed during this study are included in this article and its supplementary information files.
