# Supplementary material for: Noninvasive prenatal testing for β-thalassemia by targeted nanopore sequencing combined with relative haplotype dosage (RHDO): a feasibility study
Source: Sci Rep. 2021 Mar 11;11:5714. doi: 10.1038/s41598-021-85128-2 (PMC7952549; doi:10.1038/s41598-021-85128-2)
Supplement: Supplementary file 1 — Supplementary Legends. [file 41598_2021_85128_MOESM1_ESM.pdf]

Noninvasive prenatal testing for β-thalassemia by targeted nanopore sequencing combined with relative haplotype dosage (RHDO): a feasibility study

Fuman Jiang[1,3#], Weiqiang Liu[2#], Longmei Zhang[4 #], Yulai Guo[4], Min Chen[2], Xiaojing Zeng[4],Yang Wang[4], Yufan Li[2], JiaJia Xian[2], BoLe Du[4], Yuhuan Xie[2], Shuming Ouyang[2], Sheng Li[4], Yinghong Yang[2], Chunsheng Zhang[4], Fei Luo[1], Xiaofang Sun[2]*

[1]*School of Automation Science & Engineering, South China University of Technology, Guangzhou, 510641, China*
[2]*Department of Fetal Medicine and Prenatal Diagnosis, Key Laboratory for Major Obstetric Diseases of Guangdong Province, Key Laboratory of Reproduction and Genetics of Guangdong Higher Education Institutes, the Third Affiliated Hospital of Guangzhou Medical University, Guangzhou, 510150, China.*
[3]*Shenzhen Jingke Gene technology Co., Ltd, Shenzhen, 518052, China*
[4]*Guangzhou Jingke Medical Laboratory, Guangzhou, 510320, China*

[#]These authors contributed equally to this work.

*Corresponding Author:
Xiaofang Sun, Prof.
The Third Affiliated Hospital of Guangzhou Medical University, 63 Duobao Rd.
Guangzhou 510150, China.
Tel: 86-020-81290072
Email: xiaofangsun@gzhmu.edu.cn

Figure legends

Supplementary Figure 1. Comparison of the SNP count between nanopore sequencing and NGS data is shown for all 13 families using 10 kb (a) and 20 kb (b) libraries, respectively.

Supplementary Figure 2. The genotype in genomic position 5270539 (a) and 5248842 (b) of chr11 is validated by Sanger sequencing. The forward and reverse reads were showed respectively.

Supplementary Figure 3. The logarithmic odds ratios for fetal haplotype analysis. The fetal inherited haplotypes for 13 families are depicted using 10 kb (a) and 20 kb (b) libraries, respectively. An odds ratio less than zero indicates that the fetus inherited the maternal wild-type haplotype (Hap 1), whereas a value of more than zero indicates inheritance of the mutant haplotype (Hap 0). The red lines indicate the inheritance of paternal haplotype by the fetus. The blue lines indicate the inheritance of maternal haplotype by the fetus. The yellow stars indicate the inheritance of the mutation site by the fetus.
