# Supplementary figures and images for: Noninvasive prenatal testing for β-thalassemia by targeted nanopore sequencing combined with relative haplotype dosage (RHDO): a feasibility study
Source: Sci Rep. 2021 Mar 11;11:5714. doi: 10.1038/s41598-021-85128-2 (PMC7952549; doi:10.1038/s41598-021-85128-2)

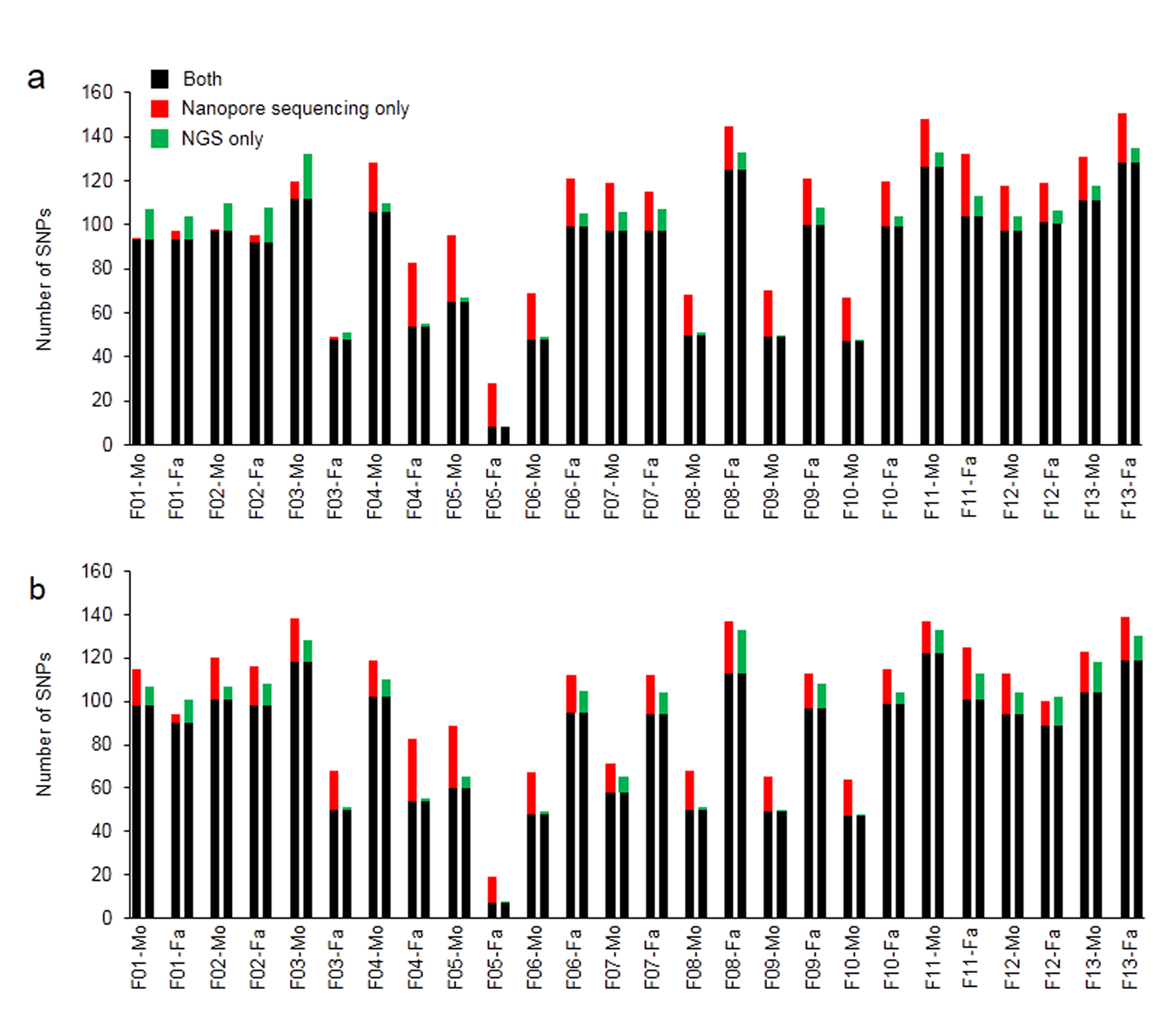

Supplement: Supplementary file 2 — Supplementary Figure S1. [file 41598_2021_85128_MOESM2_ESM.tif]

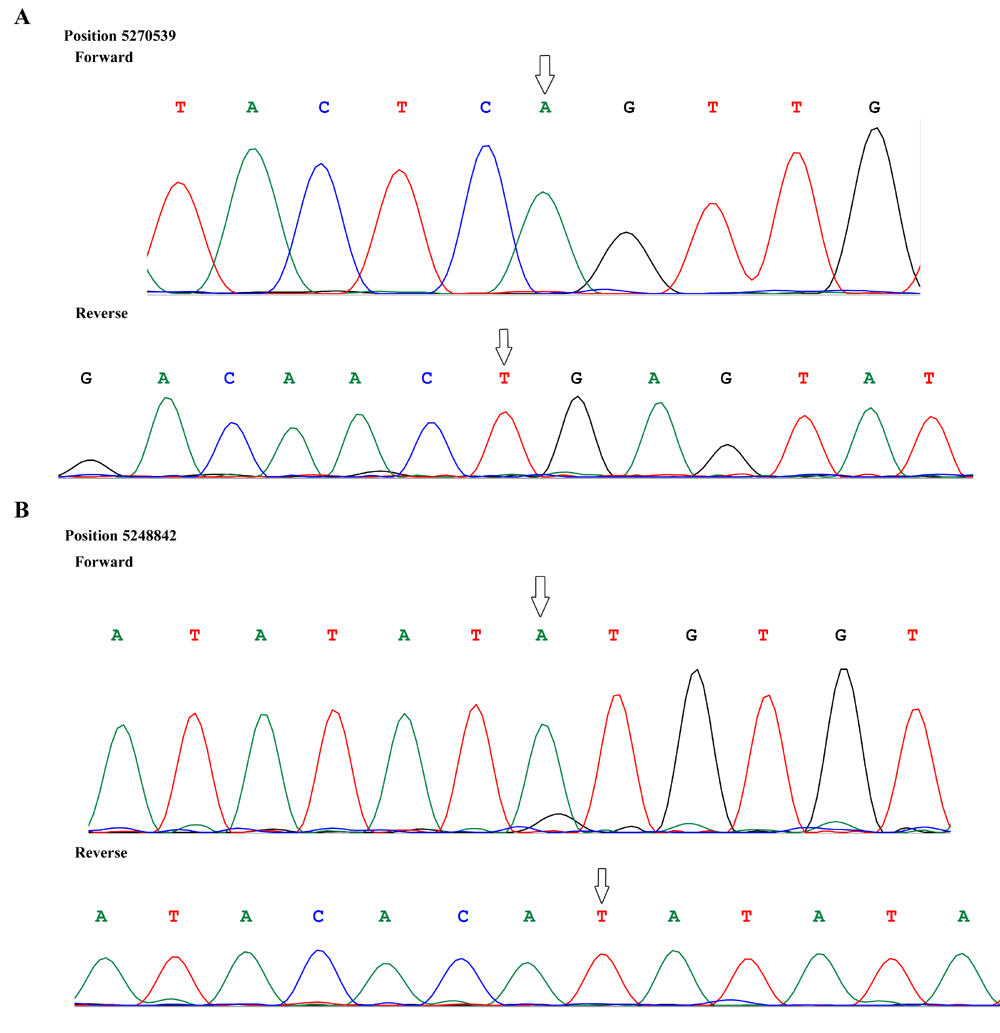

Supplement: Supplementary file 3 — Supplementary Figure S2. [file 41598_2021_85128_MOESM3_ESM.tif]
