# Supplementary material for: Noninvasive prenatal testing for β-thalassemia by targeted nanopore sequencing combined with relative haplotype dosage (RHDO): a feasibility study
Source: Sci Rep. 2021 Mar 11;11:5714. doi: 10.1038/s41598-021-85128-2 (PMC7952549; doi:10.1038/s41598-021-85128-2)
Supplement: Supplementary file 5 — Supplementary Tables. [file 41598_2021_85128_MOESM5_ESM.pdf]

Noninvasive prenatal testing for β-thalassemia by targeted nanopore sequencing combined with relative haplotype dosage (RHDO): a feasibility study

Fuman Jiang[1,3#], Weiqiang Liu[2#], Longmei Zhang[4 #], Yulai Guo[4], Min Chen[2], Xiaojing Zeng[4],Yang Wang[4], Yufan Li[2], JiaJia Xian[2], BoLe Du[4], Yuhuan Xie[2], Shuming Ouyang[2], Sheng Li[4], Yinghong Yang[2], Chunsheng Zhang[4], Fei Luo[1], Xiaofang Sun[2]*

[1]*School of Automation Science & Engineering, South China University of Technology, Guangzhou, 510641, China*
[2]*Department of Fetal Medicine and Prenatal Diagnosis, Key Laboratory for Major Obstetric Diseases of Guangdong Province, Key Laboratory of Reproduction and Genetics of Guangdong Higher Education Institutes, the Third Affiliated Hospital of Guangzhou Medical University, Guangzhou, 510150, China.*
[3]*Shenzhen Jingke Gene technology Co., Ltd, Shenzhen, 518052, China*
[4]*Guangzhou Jingke Medical Laboratory, Guangzhou, 510320, China*

[#]These authors contributed equally to this work.

*Corresponding Author:
Xiaofang Sun, Prof.
The Third Affiliated Hospital of Guangzhou Medical University, 63 Duobao Rd. Guangzhou 510150, China.
Tel: 86-020-81290072
Email: xiaofangsun@gzhmu.edu.cn

Supplemetary Table S1: Summary of MinION sequencing statistics of 10 kb amplicons.

Family		Mean read length (bp)	N50 length (bp)	Total reads	Ratio of mapped reads (%)	Sequencing depth	Ratio of >500 ×Sequencing depth (%)
F01	Mother	7203	8920	154053	99.20	20397.90	97.62
	Father	7388	9068	64315	98.98	8605.59	97.57
F02	Mother	7649	9090	57553	99.21	8061.52	97.59
	Father	7498	9069	149320	99.19	20548.17	97.62
F03	Mother	7350	9074	121706	99.01	16619.90	97.62
	Father	7440	9058	139138	99.00	19181.76	97.62
F04	Mother	8466	9076	35160	99.38	5617.16	95.05
	Father	8347	9058	36868	99.19	5745.64	95.25
F05	Mother	8234	9003	33923	99.10	5213.54	95.04
	Father	8332	9023	32762	99.40	5162.60	95.05
F06	Mother	8041	9028	30080	99.22	4540.28	95.02
	Father	8128	9064	36406	99.18	5515.64	95.04
F07	Mother	8337	9053	30685	99.37	4774.73	95.03
	Father	8298	9056	27465	99.40	4277.88	94.81
F08	Mother	8055	9043	25294	99.27	3862.64	94.76
	Father	8022	8942	30415	99.29	4596.45	93.81
F09	Mother	8367	9042	27603	99.34	4270.05	95.02
	Father	8616	9080	25479	99.63	4089.60	95.02
F10	Mother	8374	9067	41233	99.32	6468.11	97.53
	Father	8272	9048	41741	99.19	6381.27	97.57
F11	Mother	8374	9072	36425	99.41	5669.60	95.04
	Father	8436	9093	37240	99.40	5886.13	95.04
F12	Mother	8135	9046	34669	99.29	5265.08	95.00
	Father	8013	8970	29625	99.20	4403.64	95.04
F13	Mother	8177	9023	43445	99.29	6604.68	95.04
	Father	8205	9044	36449	99.22	5568.26	95.04

Supplemetary Table S2: Summary of MinION sequencing statistics of 20 kb amplicons.

Family		Mean read length (bp)	N50 length (bp)	Total reads	Ratio of mapped reads (%)	Sequencing depth	Ratio of >200×Sequencing depth (%)
F01	Mother	5183	9974	26827	97.80	2785.06	95.06
	Father	13740	16376	8818	99.40	2425.28	99.98
F02	Mother	14059	17464	7971	99.76	2273.27	99.98
	Father	7147	12307	9917	98.90	1433.79	94.88
F03	Mother	13685	16286	14575	99.60	3918.15	100.00
	Father	6939	11997	33995	97.77	3247.75	95.01
F04	Mother	6730	11923	41645	98.40	5503.04	87.06
	Father	13947	16527	7892	99.66	2243.77	99.99
F05	Mother	13201	16198	15239	99.42	3944.91	100.00
	Father	4349	7733	17754	97.56	1485.53	84.69
F06	Mother	13315	16205	11645	99.56	3071.04	100.00
	Father	5439	12386	26322	97.13	2698.77	95.05
F07	Mother	13293	17600	5649	99.58	1939.15	61.54
	Father	13258	16409	2092	99.71	557.14	98.68
F08	Mother	7515	15126	20111	97.68	2835.02	95.03
	Father	7108	15534	24666	98.38	3468.16	95.04
F09	Mother	8587	14926	5193	97.90	795.61	94.96
	Father	7631	15585	1443	97.85	212.22	53.32
F10	Mother	12180	16572	7762	99.34	1681.47	95.05
	Father	7639	15182	7419	96.47	1099.48	94.99
F11	Mother	6117	11445	15860	97.85	1800.27	95.03
	Father	5634	12920	1979	95.81	217.86	53.11
F12	Mother	6772	14888	38731	97.63	5303.78	86.36
	Father	13502	16028	11371	99.48	3074.98	96.79
F13	Mother	3451	6821	30973	94.87	2105.60	91.99
	Father	13622	16311	2818	99.65	780.65	99.56

Supplemetary Table S3. Comparison between MinION sequencing data and NGS data of 10 kb amplicons.

Family		Number of SNPs detected only by nanopore sequencing	Number of SNPs detected only by NGS	Number of SNPs detected by both methods	Number of the consistent SNPs detected by both methods	Number of heterozygous SNPs detected by both methods
F01	Mother	1	14	93	90	49
	Father	4	11	93	90	51
F02	Mother	1	13	97	96	57
	Father	3	16	92	91	51
F03	Mother	8	20	112	112	41
	Father	1	3	48	47	6
F04	Mother	22	4	106	106	103
	Father	29	1	54	52	40
F05	Mother	30	2	65	62	50
	Father	20	0	8	6	6
F06	Mother	21	1	48	47	5
	Father	22	6	99	97	54
F07	Mother	22	9	97	97	54
	Father	18	10	97	97	95
F08	Mother	18	1	50	48	5
	Father	20	8	125	120	47
F09	Mother	21	1	49	47	5
	Father	21	8	100	99	56
F10	Mother	20	1	47	45	4
	Father	21	5	99	98	56
F11	Mother	22	7	126	123	51
	Father	28	9	104	101	89
F12	Mother	21	7	97	96	54
	Father	18	5	101	100	5
F13	Mother	20	7	111	109	50
	Father	23	7	128	126	51

Supplemetary Table S4. Comparison between MinION sequencing data and NGS data of 20 kb amplicons.

Family		Number of SNPs detected only by nanopore sequencing	Number of SNPs detected only by NGS	Number of SNPs detected by both methods	Number of the consistent SNPs detected by both methods	Number of heterozygous SNPs detected by both methods
F01	Mother	17	9	98	96	53
	Father	4	11	90	87	48
F02	Mother	19	6	101	100	59
	Father	18	10	98	96	53
F03	Mother	20	10	118	117	47
	Father	18	1	50	49	6
F04	Mother	17	8	102	102	100
	Father	29	1	54	52	40
F05	Mother	29	5	60	58	47
	Father	12	1	7	5	5
F06	Mother	19	1	48	47	6
	Father	17	10	95	93	50
F07	Mother	13	7	58	58	32
	Father	18	10	94	94	92
F08	Mother	18	1	50	48	5
	Father	24	20	113	103	39
F09	Mother	16	1	49	47	5
	Father	16	11	97	96	53
F10	Mother	17	1	47	45	4
	Father	16	5	99	98	56
F11	Mother	15	11	122	119	49
	Father	24	12	101	98	86
F12	Mother	19	10	94	93	51
	Father	11	13	89	88	6
F13	Mother	19	14	104	103	43
	Father	20	11	119	118	47

Supplemetary Table S5: Characteristic of plasma sequencing data.

Family	Raw data (Mb)	Total reads	Coverage of target region (%)	Sequencing depth	Ratio of >100×Sequencing depth (%)	Fetal fraction (%)
F01	2245.57	15237739	99.47	613.99	93.45	12
F02	3142.48	21475586	99.47	895.13	94.29	20
F03	1527.16	10519074	96.87	490.13	87.72	17
F04	2085.37	14236162	99.45	539.83	93.07	13
F05	2509.49	17336363	99.39	664.83	93.49	18
F06	2485.34	17111470	99.48	331.21	90.88	20
F07	2500.62	17027931	99.34	362.25	90.93	14
F08	867.11	5780725	99.57	321.43	90.19	8
F09	2439.36	16636840	99.38	670.89	93.78	12
F10	2435	16550285	99.32	339.39	90.80	10
F11	2784.7	19127744	99.52	416.39	92.01	19
F12	834.36	5562430	99.54	317.05	92.53	9
F13	2479.48	16948623	99.39	379.25	90.63	11

Supplemetary Table S6: Determining the paternally and maternally inherited haplotype by RHDO analysis of 10 kb amplicons.

Family			Genomic location	Counts for the allele on Hap 0	Counts for the allele on Hap 1	Cumulat-ive count for alleles on Hap 0	Cumulat-ive count for alleles on Hap 1	Total no. of reads	Fraction of total reads contributed by Hap 0 alleles	Classification threshold (upper, lower)	SPRT classification result
F01	Father	type α SNPs	5223970	890	9	890	9	899	0.9900	(0.9835, 0.9872)	Hap 0
			5228143	1013	0	1013	0	1013	1.0000	(0.9837, 0.9870)	Hap 0
			5228708	993	3	993	3	996	0.9970	(0.9836, 0.9871)	Hap 0
			5229196	884	3	884	3	887	0.9966	(0.9834, 0.9873)	Hap 0
			5233447	24	0	24	0	24	1.0000	(0.9143, 1.0564)	Unclassified
			5234781	148	0	172	0	172	1.0000	(0.9754, 0.9953)	Hap 0
			5236851	947	2	947	2	949	0.9979	(0.9836, 0.9871)	Hap 0
			5243559	89	0	89	0	89	1.0000	(0.9662, 1.0045)	Unclassified
			5243613	211	0	300	0	300	1.0000	(0.9797, 0.9910)	Hap 0
			5244404	572	1	572	1	573	0.9983	(0.9824, 0.9883)	Hap 0
			5246356	822	1	822	1	823	0.9988	(0.9833, 0.9874)	Hap 0
		type β SNPs	5247992	45	990	45	990	1035	0.0435	(0.0197, 0.0251)	Hap 0
			5257189	47	882	47	882	929	0.0506	(0.0194, 0.0254)	Hap 0
			5259000	43	1010	43	1010	1053	0.0408	(0.0197, 0.0250)	Hap 0
	Mather	type α SNPs	5229835	342	300	342	300	642	0.5327	(0.4843, 0.5759)	Unclassified
			5237435	13	11	355	311	666	0.5330	(0.4859,	Unclassified

										0.5742)	
			5246514	471	331	826	642	1468	0.5627	(0.5100, 0.5501)	Hap 0
		type β SNPs	5223871	517	492	517	492	1009	0.5124	(0.4408, 0.4991)	Hap 0
			5224054	431	476	431	476	907	0.4752	(0.4375, 0.5023)	Unclassified
			5226579	40	67	471	543	1014	0.4645	(0.4409, 0.4989)	Unclassified
			5227471	355	360	826	903	1729	0.4777	(0.4529, 0.4869)	Unclassified
			5229010	482	459	1308	1362	2670	0.4899	(0.4589, 0.4809)	Hap 0
			5232212	35	48	35	48	83	0.4217	(0.1157, 0.8241)	Unclassified
			5233697	134	104	169	152	321	0.5265	(0.3783, 0.5615)	Unclassified
			5233836	364	335	533	487	1020	0.5225	(0.4411, 0.4988)	Hap 0
			5234029	9	10	9	10	19	0.4737	(-1.0774, 2.0173)	Unclassified
			5234542	25	37	34	47	81	0.4198	(0.1070, 0.8329)	Unclassified
			5236740	271	275	305	322	627	0.4864	(0.4230, 0.5168)	Unclassified
			5237284	117	145	422	467	889	0.4747	(0.4369, 0.5030)	Unclassified
			5244144	398	393	820	860	1680	0.4881	(0.4524, 0.4874)	Hap 0
			5246000	408	368	408	368	776	0.5258	(0.4320, 0.5078)	Hap 0
			5246203	394	324	394	324	718	0.5487	(0.4290, 0.5109)	Hap 0
			5247153	338	387	338	387	725	0.4662	(0.4294, 0.5105)	Unclassified
			5247733	439	464	777	851	1628	0.4773	(0.4519, 0.4880)	Unclassified
F02	Father	type α SNPs	5229835	897	4	897	4	901	0.9956	(0.9762, 0.9795)	Hap 0
			5237435	14	0	14	0	14	1.0000	(0.8703, 1.0854)	Unclassified
			5246514	1361	2	1375	2	1377	0.9985	(0.9767,	Hap 0

										0.9789)	
		type β SNPs	5224054	140	1206	140	1206	1346	0.1040	(0.0307, 0.0341)	Hap 0
			5226579	2	73	2	73	75	0.0267	(0.0019, 0.0629)	Unclassified
			5227471	97	842	99	915	1014	0.0976	(0.0302, 0.0347)	Hap 0
			5229010	152	1437	152	1437	1589	0.0957	(0.0310, 0.0339)	Hap 0
			5232212	3	88	3	88	91	0.0330	(0.0072, 0.0576)	Unclassified
			5233697	25	298	28	386	414	0.0676	(0.0269, 0.0379)	Hap 0
			5233836	126	944	126	944	1070	0.1178	(0.0303, 0.0346)	Hap 0
			5234029	1	22	1	22	23	0.0435	(-0.0672, 0.1320)	Unclassified
			5234542	3	61	4	83	87	0.0460	(0.0061, 0.0587)	Unclassified
			5236740	62	663	66	746	812	0.0813	(0.0296, 0.0352)	Hap 0
			5237284	29	299	29	299	328	0.0884	(0.0254, 0.0394)	Hap 0
			5244144	70	938	70	938	1008	0.0694	(0.0301, 0.0347)	Hap 0
			5246000	138	1350	138	1350	1488	0.0927	(0.0309, 0.0340)	Hap 0
			5246203	139	1135	139	1135	1274	0.1091	(0.0306, 0.0342)	Hap 0
			5247153	94	962	94	962	1056	0.0890	(0.0302, 0.0346)	Hap 0
			5247733	165	1454	165	1454	1619	0.1019	(0.0310, 0.0338)	Hap 0
	Mather	type α SNPs	5228143	845	533	845	533	1378	0.6132	(0.5377, 0.5630)	Hap 0
			5228708	922	559	922	559	1481	0.6226	(0.5385, 0.5621)	Hap 0
			5229196	728	434	728	434	1162	0.6265	(0.5353, 0.5654)	Hap 0
			5233447	6	4	6	4	10	0.6000	(-1.1983, 2.2990)	Unclassified
			5234781	133	88	139	92	231	0.6017	(0.4746,	Unclassified

										0.6260)	
			5236851	822	558	961	650	1611	0.5965	(0.5395, 0.5612)	Hap 0
			5243559	67	45	67	45	112	0.5982	(0.3942, 0.7065)	Unclassified
			5243613	142	116	209	161	370	0.5649	(0.5031, 0.5976)	Unclassified
			5244383	464	264	673	425	1098	0.6129	(0.5344, 0.5663)	Hap 0
			5244404	427	237	427	237	664	0.6431	(0.5240, 0.5767)	Hap 0
			5246356	947	513	947	513	1460	0.6486	(0.5384, 0.5623)	Hap 0
			5248852	328	48	328	48	376	0.8723	(0.5038, 0.5968)	Hap 0
		type β SNPs	5248200	726	777	726	777	1503	0.4830	(0.4380, 0.4613)	Hap 0
			5248770	517	331	517	331	848	0.6097	(0.4290, 0.4703)	Hap 0
			5248842	286	54	286	54	340	0.8412	(0.3982, 0.5011)	Hap 0
F03	Father	type β SNPs	5247992	86	616	86	616	702	0.1225	(0.0253, 0.0322)	Hap 0
			5257189	52	326	52	326	378	0.1376	(0.0223, 0.0352)	Hap 0
			5259000	41	399	41	399	440	0.0932	(0.0232, 0.0343)	Hap 0
	Mather	type α SNPs	5223970	203	80	203	80	283	0.7173	(0.4697, 0.6157)	Hap 0
			5228708	235	148	235	148	383	0.6136	(0.4888, 0.5966)	Hap 0
			5229196	210	101	210	101	311	0.6752	(0.4763, 0.6091)	Hap 0
			5236851	216	102	216	102	318	0.6792	(0.4778, 0.6076)	Hap 0
			5243613	34	13	34	13	47	0.7234	(0.1033, 0.9821)	Unclassified
			5244404	126	58	160	71	231	0.6926	(0.4533, 0.6321)	Hap 0
			5246356	232	98	232	98	330	0.7030	(0.4801, 0.6053)	Hap 0

		type β SNPs	5223871	161	225	161	225	386	0.4171	(0.4038, 0.5108)	Unclassified
			5224054	137	190	298	415	713	0.4180	(0.4283, 0.4863)	Hap 1
			5227471	104	114	104	114	218	0.4771	(0.3626, 0.5520)	Unclassified
			5229010	215	230	319	344	663	0.4811	(0.4261, 0.4884)	Unclassified
			5233697	16	24	335	368	703	0.4765	(0.4279, 0.4867)	Unclassified
			5233836	122	154	457	522	979	0.4668	(0.4362, 0.4784)	Unclassified
			5234542	4	6	461	528	989	0.4661	(0.4364, 0.4782)	Unclassified
			5236740	96	89	557	617	1174	0.4744	(0.4397, 0.4749)	Unclassified
			5237284	38	18	595	635	1230	0.4837	(0.4405, 0.4741)	Hap 0
			5244144	102	111	102	111	213	0.4789	(0.3603, 0.5542)	Unclassified
			5246000	209	213	311	324	635	0.4898	(0.4248, 0.4898)	Unclassified
			5246203	149	179	460	503	963	0.4777	(0.4358, 0.4787)	Unclassified
			5247153	143	136	603	639	1242	0.4855	(0.4407, 0.4739)	Hap 0
			5247733	205	232	205	232	437	0.4691	(0.4100, 0.5045)	Unclassified
F04	Father	type α SNPs	5255989	818	0	818	0	818	1.0000	(0.9823, 0.9864)	Hap 0
			5257778	837	0	837	0	837	1.0000	(0.9824, 0.9864)	Hap 0
			5263683	636	0	636	0	636	1.0000	(0.9818, 0.9870)	Hap 0
			5264146	903	2	903	2	905	0.9978	(0.9825, 0.9862)	Hap 0
			5265106	826	0	826	0	826	1.0000	(0.9824, 0.9864)	Hap 0
			5265680	406	0	406	0	406	1.0000	(0.9803, 0.9885)	Hap 0
			5269931	690	1	690	1	691	0.9986	(0.9820, 0.9868)	Hap 0

			5271671	867	4	867	4	871	0.9954	(0.9825, 0.9863)	Hap 0
		type β SNPs	5248200	42	800	42	800	842	0.0499	(0.0205, 0.0269)	Hap 0
			5248842	20	136	20	136	156	0.1282	(0.0064, 0.0410)	Hap 0
	Mather	type α SNPs	5223920	406	300	406	300	706	0.5751	(0.4942, 0.5710)	Hap 0
			5223970	377	256	377	256	633	0.5956	(0.4898, 0.5754)	Hap 0
			5224660	369	295	369	295	664	0.5557	(0.4918, 0.5734)	Unclassified
			5227950	327	214	696	509	1205	0.5776	(0.5101, 0.5551)	Hap 0
			5227975	326	214	326	214	540	0.6037	(0.4824, 0.5828)	Hap 0
			5228143	458	398	458	398	856	0.5350	(0.5009, 0.5643)	Unclassified
			5228708	457	377	915	775	1690	0.5414	(0.5165, 0.5486)	Unclassified
			5229196	481	336	1396	1111	2507	0.5568	(0.5218, 0.5434)	Hap 0
			5229743	409	156	409	156	565	0.7239	(0.4846, 0.5806)	Hap 0
			5229745	410	151	410	151	561	0.7308	(0.4843, 0.5809)	Hap 0
			5231897	515	377	515	377	892	0.5774	(0.5022, 0.5630)	Hap 0
			5232146	37	28	37	28	65	0.5692	(0.1154, 0.9497)	Unclassified
			5232573	425	344	462	372	834	0.5540	(0.5001, 0.5651)	Unclassified
			5233447	12	10	474	382	856	0.5537	(0.5009, 0.5643)	Unclassified
			5233643	29	28	503	410	913	0.5509	(0.5029, 0.5623)	Unclassified
			5234587	61	28	564	438	1002	0.5629	(0.5055, 0.5597)	Hap 0
			5234781	102	77	102	77	179	0.5698	(0.3811, 0.6841)	Unclassified
			5236417	526	403	628	480	1108	0.5668	(0.5081, 0.5571)	Hap 0

			5236851	475	369	475	369	844	0.5628	(0.5005, 0.5647)	Unclassified
			5243559	48	44	523	413	936	0.5588	(0.5036, 0.5616)	Unclassified
			5243613	95	104	618	517	1135	0.5445	(0.5087, 0.5565)	Unclassified
			5243757	361	309	979	826	1805	0.5424	(0.5176, 0.5476)	Unclassified
			5244299	209	161	1188	987	2175	0.5462	(0.5201, 0.5451)	Hap 0
			5244404	284	179	284	179	463	0.6134	(0.4740, 0.5912)	Hap 0
			5245507	346	273	346	273	619	0.5590	(0.4888, 0.5764)	Unclassified
			5246042	416	323	762	596	1358	0.5611	(0.5126, 0.5526)	Hap 0
			5246356	532	342	532	342	874	0.6087	(0.5016, 0.5636)	Hap 0
			5246512	368	338	368	338	706	0.5212	(0.4942, 0.5710)	Unclassified
			5247141	362	280	730	618	1348	0.5415	(0.5125, 0.5527)	Unclassified
			5247791	512	480	1242	1098	2340	0.5308	(0.5210, 0.5442)	Unclassified
			5248243	403	376	1645	1474	3119	0.5274	(0.5239, 0.5413)	Unclassified
			5248641	416	347	2061	1821	3882	0.5309	(0.5256, 0.5396)	Unclassified
			5248770	257	205	2318	2026	4344	0.5336	(0.5264, 0.5388)	Unclassified
			5248852	170	96	2488	2122	4610	0.5397	(0.5267, 0.5385)	Hap 0
			5249715	351	154	351	154	505	0.6950	(0.4789, 0.5863)	Hap 0
			5257189	455	359	455	359	814	0.5590	(0.4993, 0.5659)	Unclassified
			5259000	499	305	954	664	1618	0.5896	(0.5158, 0.5494)	Hap 0
			5265941	436	324	436	324	760	0.5737	(0.4969, 0.5683)	Hap 0
		type β SNPs	5248329	400	431	400	431	831	0.4813	(0.4348, 0.5000)	Unclassified
			5249370	309	354	709	785	1494	0.4746	(0.4493,	Unclassified

										0.4856)	
			5256431	365	318	1074	1103	2177	0.4933	(0.4550, 0.4799)	Hap 0
			5259534	39	15	39	15	54	0.7222	(-0.0347, 0.9695)	Unclassified
			5262782	438	469	477	484	961	0.4964	(0.4392, 0.4956)	Hap 0
			5263577	390	408	390	408	798	0.4887	(0.4334, 0.5014)	Unclassified
			5264929	437	312	827	720	1547	0.5346	(0.4499, 0.4849)	Hap 0
			5265366	66	52	66	52	118	0.5593	(0.2376, 0.6972)	Unclassified
			5265475	8	4	74	56	130	0.5692	(0.2588, 0.6760)	Unclassified
			5266961	378	368	452	424	876	0.5160	(0.4365, 0.4984)	Hap 0
			5269140	593	512	593	512	1105	0.5367	(0.4429, 0.4919)	Hap 0
			5269799	292	295	292	295	587	0.4974	(0.4212, 0.5136)	Unclassified
			5270418	477	419	769	714	1483	0.5185	(0.4491, 0.4857)	Hap 0
F05	Father	type α SNPs	5255044	544	1	544	1	545	0.9982	(0.9769, 0.9825)	Hap 0
		type β SNPs	5248329	106	835	106	835	941	0.1126	(0.0274, 0.0325)	Hap 0
			5249004	73	709	73	709	782	0.0934	(0.0269, 0.0330)	Hap 0
	Mather	type α SNPs	5223871	559	502	559	502	1061	0.5269	(0.5269, 0.5636)	Hap 1
			5231897	518	523	518	523	1041	0.4976	(0.5265, 0.5640)	Hap 1
			5232212	38	17	38	17	55	0.6909	(0.1911, 0.8994)	Unclassified
			5232573	503	482	541	499	1040	0.5202	(0.5265, 0.5640)	Hap 1
			5233643	28	31	28	31	59	0.4746	(0.2151, 0.8754)	Unclassified
			5233697	120	131	148	162	310	0.4774	(0.4824, 0.6081)	Hap 1

			5234029	6	6	6	6	12	0.5000	(-1.0781, 2.1686)	Unclassified
			5234542	27	29	33	35	68	0.4853	(0.2588, 0.8317)	Unclassified
			5234587	45	41	78	76	154	0.5065	(0.4188, 0.6717)	Unclassified
			5243856	465	412	543	488	1031	0.5267	(0.5264, 0.5641)	Unclassified
			5257778	450	507	993	995	1988	0.4995	(0.5354, 0.5550)	Hap 1
			5263683	452	370	452	370	822	0.5499	(0.5215, 0.5689)	Unclassified
			5264146	511	471	963	841	1804	0.5338	(0.5344, 0.5560)	Hap 1
			5265106	511	531	511	531	1042	0.4904	(0.5266, 0.5639)	Hap 1
			5265680	254	253	254	253	507	0.5010	(0.5068, 0.5837)	Hap 1
			5269931	420	305	420	305	725	0.5793	(0.5184, 0.5721)	Hap 0
			5271671	563	472	563	472	1035	0.5440	(0.5264, 0.5641)	Unclassified
		type β SNPs	5247992	383	745	383	745	1128	0.3395	(0.4375, 0.4720)	Hap 1
			5250168	302	377	302	377	679	0.4448	(0.4261, 0.4834)	Unclassified
			5252251	222	423	524	800	1324	0.3958	(0.4400, 0.4695)	Hap 1
			5253477	208	376	208	376	584	0.3562	(0.4214, 0.4881)	Hap 1
			5255912	288	421	288	421	709	0.4062	(0.4273, 0.4822)	Hap 1
			5256647	249	394	249	394	643	0.3872	(0.4245, 0.4850)	Hap 1
			5258038	307	485	307	485	792	0.3876	(0.4302, 0.4793)	Hap 1
			5258162	197	344	197	344	541	0.3641	(0.4187, 0.4908)	Hap 1
			5258265	33	79	33	79	112	0.2946	(0.2808, 0.6287)	Unclassified
			5258429	136	250	169	329	498	0.3394	(0.4156, 0.4939)	Hap 1
			5258490	199	450	199	450	649	0.3066	(0.4247,	Hap 1

										0.4848)	
			5258592	367	639	367	639	1006	0.3648	(0.4354, 0.4741)	Hap 1
			5258827	393	637	393	637	1030	0.3816	(0.4358, 0.4737)	Hap 1
			5258852	418	670	418	670	1088	0.3842	(0.4368, 0.4727)	Hap 1
			5258856	415	673	415	673	1088	0.3814	(0.4368, 0.4727)	Hap 1
			5258989	345	593	345	593	938	0.3678	(0.4340, 0.4755)	Hap 1
			5259103	352	666	352	666	1018	0.3458	(0.4356, 0.4739)	Hap 1
			5259289	157	281	157	281	438	0.3584	(0.4103, 0.4992)	Hap 1
			5259292	155	276	155	276	431	0.3596	(0.4096, 0.5000)	Hap 1
			5259419	22	40	22	40	62	0.3548	(0.1406, 0.7689)	Unclassified
			5259727	320	405	342	445	787	0.4346	(0.4300, 0.4795)	Unclassified
			5260458	363	604	705	1049	1754	0.4019	(0.4436, 0.4659)	Hap 1
			5260576	364	559	364	559	923	0.3944	(0.4336, 0.4759)	Hap 1
			5261239	413	601	413	601	1014	0.4073	(0.4355, 0.4740)	Hap 1
			5266728	395	608	395	608	1003	0.3938	(0.4353, 0.4742)	Hap 1
			5268406	377	593	377	593	970	0.3887	(0.4347, 0.4748)	Hap 1
			5268622	398	623	398	623	1021	0.3898	(0.4357, 0.4738)	Hap 1
			5268797	430	607	430	607	1037	0.4147	(0.4360, 0.4735)	Hap 1
			5269343	460	734	460	734	1194	0.3853	(0.4384, 0.4711)	Hap 1
			5269806	264	407	264	407	671	0.3934	(0.4257, 0.4838)	Hap 1
			5270246	399	405	399	405	804	0.4963	(0.4305, 0.4790)	Hap 0
			5270343	380	177	380	177	557	0.6822	(0.4198, 0.4897)	Hap 0

			5272154	461	742	461	742	1203	0.3832	(0.4386, 0.4709)	Hap 1
F06	Father	type α SNPs	5229835	147	0	147	0	147	1.0000	(0.9700, 0.9912)	Hap 0
			5237435	25	0	25	0	25	1.0000	(0.9180, 1.0432)	Unclassified
			5246514	418	0	443	0	443	1.0000	(0.9771, 0.9841)	Hap 0
		type β SNPs	5223750	6	192	6	192	198	0.0303	(0.0165, 0.0410)	Unclassified
			5223871	17	282	23	474	497	0.0463	(0.0239, 0.0336)	Hap 0
			5224054	4	188	4	188	192	0.0208	(0.0161, 0.0414)	Unclassified
			5224660	7	239	11	427	438	0.0251	(0.0232, 0.0343)	Unclassified
			5226579	0	11	11	438	449	0.0245	(0.0233, 0.0342)	Unclassified
			5227471	15	197	26	635	661	0.0393	(0.0251, 0.0324)	Hap 0
			5227950	3	135	3	135	138	0.0217	(0.0111, 0.0464)	Unclassified
			5227975	5	148	8	283	291	0.0275	(0.0204, 0.0371)	Unclassified
			5229010	29	348	37	631	668	0.0554	(0.0251, 0.0324)	Hap 0
			5229743	11	244	11	244	255	0.0431	(0.0192, 0.0383)	Hap 0
			5229745	10	248	10	248	258	0.0388	(0.0193, 0.0382)	Hap 0
			5230302	23	442	23	442	465	0.0495	(0.0235, 0.0340)	Hap 0
			5231897	29	471	29	471	500	0.0580	(0.0239, 0.0336)	Hap 0
			5232146	2	67	2	67	69	0.0290	(-0.0065, 0.0640)	Unclassified
			5232212	3	72	5	139	144	0.0347	(0.0119, 0.0456)	Unclassified
			5232573	27	322	32	461	493	0.0649	(0.0238, 0.0337)	Hap 0
			5233643	13	209	13	209	222	0.0586	(0.0178, 0.0397)	Hap 0

			5233697	11	314	11	314	325	0.0338	(0.0213, 0.0362)	Unclassified
			5233836	32	398	43	712	755	0.0570	(0.0255, 0.0320)	Hap 0
			5234029	28	421	28	421	449	0.0624	(0.0233, 0.0342)	Hap 0
			5234542	0	21	0	21	21	0.0000	(-0.0870, 0.1445)	Unclassified
			5234587	0	13	0	34	34	0.0000	(-0.0427, 0.1002)	Unclassified
			5236417	24	424	24	458	482	0.0498	(0.0237, 0.0338)	Hap 0
			5236740	29	381	29	381	410	0.0707	(0.0228, 0.0347)	Hap 0
			5237284	6	159	6	159	165	0.0364	(0.0140, 0.0435)	Unclassified
			5237498	0	14	6	173	179	0.0335	(0.0152, 0.0423)	Unclassified
			5243757	14	209	20	382	402	0.0498	(0.0227, 0.0348)	Hap 0
			5244144	6	125	6	125	131	0.0458	(0.0102, 0.0473)	Unclassified
			5244299	9	81	15	206	221	0.0679	(0.0178, 0.0397)	Hap 0
			5245507	12	140	12	140	152	0.0789	(0.0128, 0.0447)	Hap 0
			5246000	30	303	30	303	333	0.0901	(0.0214, 0.0360)	Hap 0
			5246042	26	334	26	334	360	0.0722	(0.0220, 0.0355)	Hap 0
			5246203	52	408	52	408	460	0.1130	(0.0235, 0.0340)	Hap 0
			5246512	35	388	35	388	423	0.0827	(0.0230, 0.0345)	Hap 0
			5247141	21	242	21	242	263	0.0798	(0.0195, 0.0380)	Hap 0
			5247153	21	226	21	226	247	0.0850	(0.0189, 0.0386)	Hap 0
			5247733	28	338	28	338	366	0.0765	(0.0221, 0.0354)	Hap 0
			5247791	27	459	27	459	486	0.0556	(0.0237, 0.0337)	Hap 0
			5248243	35	406	35	406	441	0.0794	(0.0232, 0.0343)	Hap 0

			5248641	20	286	20	286	306	0.0654	(0.0208, 0.0367)	Hap 0
	Mather	type β SNPs	5248200	262	205	262	205	467	0.5610	(0.4131, 0.5015)	Hap 0
			5248842	67	58	67	58	125	0.5360	(0.2922, 0.6224)	Unclassified
F07	Father	type α SNPs	5234678	114	0	114	0	114	1.0000	(0.9690, 0.9978)	Hap 0
			5250168	395	0	395	0	395	1.0000	(0.9793, 0.9876)	Hap 0
			5252251	322	1	322	1	323	0.9969	(0.9783, 0.9885)	Hap 0
			5253477	277	0	277	0	277	1.0000	(0.9775, 0.9893)	Hap 0
			5255912	408	0	408	0	408	1.0000	(0.9794, 0.9874)	Hap 0
			5256431	542	0	542	0	542	1.0000	(0.9804, 0.9865)	Hap 0
			5256647	264	0	264	0	264	1.0000	(0.9772, 0.9896)	Hap 0
			5258038	463	0	463	0	463	1.0000	(0.9799, 0.9870)	Hap 0
			5258162	356	0	356	0	356	1.0000	(0.9788, 0.9880)	Hap 0
			5258265	75	0	75	0	75	1.0000	(0.9615, 1.0053)	Unclassified
			5258429	228	0	303	0	303	1.0000	(0.9780, 0.9888)	Hap 0
			5258490	302	0	302	0	302	1.0000	(0.9780, 0.9889)	Hap 0
			5258592	503	0	503	0	503	1.0000	(0.9802, 0.9867)	Hap 0
			5258827	543	0	543	0	543	1.0000	(0.9804, 0.9864)	Hap 0
			5258852	558	0	558	0	558	1.0000	(0.9805, 0.9864)	Hap 0
			5258856	556	0	556	0	556	1.0000	(0.9805, 0.9864)	Hap 0
			5258989	478	0	478	0	478	1.0000	(0.9800, 0.9869)	Hap 0
			5259289	227	0	227	0	227	1.0000	(0.9762, 0.9907)	Hap 0

			5259292	212	0	212	0	212	1.0000	(0.9757, 0.9912)	Hap 0
			5259419	41	0	41	0	41	1.0000	(0.9434, 1.0234)	Unclassified
			5259534	41	0	82	0	82	1.0000	(0.9634, 1.0034)	Unclassified
			5259727	374	0	456	0	456	1.0000	(0.9798, 0.9870)	Hap 0
			5260458	484	0	484	0	484	1.0000	(0.9800, 0.9868)	Hap 0
			5260576	537	0	537	0	537	1.0000	(0.9804, 0.9865)	Hap 0
			5261239	505	0	505	0	505	1.0000	(0.9802, 0.9867)	Hap 0
			5262782	628	0	628	0	628	1.0000	(0.9808, 0.9860)	Hap 0
			5263577	575	0	575	0	575	1.0000	(0.9806, 0.9863)	Hap 0
			5264929	401	0	401	0	401	1.0000	(0.9793, 0.9875)	Hap 0
			5265366	67	1	67	1	68	0.9853	(0.9593, 1.0076)	Unclassified
			5265475	11	0	78	1	79	0.9873	(0.9627, 1.0042)	Unclassified
			5266728	490	0	568	1	569	0.9982	(0.9805, 0.9863)	Hap 0
			5266961	444	0	444	0	444	1.0000	(0.9797, 0.9871)	Hap 0
			5268406	487	0	487	0	487	1.0000	(0.9801, 0.9868)	Hap 0
			5268622	613	0	613	0	613	1.0000	(0.9807, 0.9861)	Hap 0
			5268797	509	0	509	0	509	1.0000	(0.9802, 0.9866)	Hap 0
			5269140	616	1	616	1	617	0.9984	(0.9808, 0.9861)	Hap 0
			5269343	502	0	502	0	502	1.0000	(0.9802, 0.9867)	Hap 0
			5269799	345	0	345	0	345	1.0000	(0.9787, 0.9882)	Hap 0
			5269806	356	2	356	2	358	0.9944	(0.9788, 0.9880)	Hap 0
			5270418	525	0	525	0	525	1.0000	(0.9803, 0.9865)	Hap 0

			5270539	408	0	408	0	408	1.0000	(0.9794, 0.9874)	Hap 0
			5272154	593	0	593	0	593	1.0000	(0.9807, 0.9862)	Hap 0
		type β SNPs	5246514	24	462	24	462	486	0.0494	(0.0196, 0.0304)	Hap 0
			5247904	28	531	28	531	559	0.0501	(0.0203, 0.0297)	Hap 0
			5250551	27	553	27	553	580	0.0466	(0.0205, 0.0295)	Hap 0
			5253586	19	445	19	445	464	0.0409	(0.0193, 0.0306)	Hap 0
			5258336	2	55	2	55	57	0.0351	(-0.0210, 0.0709)	Unclassified
	Mather	type β SNPs	5247992	236	328	236	328	564	0.4184	(0.4203, 0.5094)	Hap 1
F08	Father	type α SNPs	5223871	363	0	363	0	363	1.0000	(0.9815, 0.9911)	Hap 0
			5224054	338	0	338	0	338	1.0000	(0.9812, 0.9915)	Hap 0
			5226579	31	0	31	0	31	1.0000	(0.9300, 1.0426)	Unclassified
			5227471	331	0	362	0	362	1.0000	(0.9815, 0.9911)	Hap 0
			5229010	480	0	480	0	480	1.0000	(0.9827, 0.9899)	Hap 0
			5232212	97	0	97	0	97	1.0000	(0.9683, 1.0043)	Unclassified
			5233697	348	0	445	0	445	1.0000	(0.9824, 0.9902)	Hap 0
			5233836	443	0	443	0	443	1.0000	(0.9824, 0.9903)	Hap 0
			5234029	470	0	470	0	470	1.0000	(0.9826, 0.9900)	Hap 0
			5234542	23	0	23	0	23	1.0000	(0.9105, 1.0622)	Unclassified
			5236740	458	0	481	0	481	1.0000	(0.9827, 0.9899)	Hap 0
			5237284	190	0	190	0	190	1.0000	(0.9771, 0.9955)	Hap 0
			5237498	25	0	25	0	25	1.0000	(0.9165,	Unclassified

										1.0561)	
			5244144	168	0	193	0	193	1.0000	(0.9773, 0.9954)	Hap 0
			5246000	449	0	449	0	449	1.0000	(0.9824, 0.9902)	Hap 0
			5246203	522	0	522	0	522	1.0000	(0.9830, 0.9897)	Hap 0
			5247733	435	0	435	0	435	1.0000	(0.9823, 0.9903)	Hap 0
		type β SNPs	5223920	7	309	7	309	316	0.0222	(0.0119, 0.0302)	Unclassified
			5223970	2	270	9	579	588	0.0153	(0.0161, 0.0260)	Hap 1
			5228143	27	507	27	507	534	0.0506	(0.0156, 0.0265)	Hap 0
			5228708	20	428	20	428	448	0.0446	(0.0146, 0.0275)	Hap 0
			5229196	30	371	30	371	401	0.0748	(0.0138, 0.0283)	Hap 0
			5233447	8	271	8	271	279	0.0287	(0.0107, 0.0314)	Unclassified
			5234781	1	20	9	291	300	0.0300	(0.0114, 0.0307)	Unclassified
			5236851	13	481	22	772	794	0.0277	(0.0174, 0.0247)	Hap 0
			5243559	3	52	3	52	55	0.0545	(-0.0315, 0.0737)	Unclassified
			5243613	9	107	12	159	171	0.0702	(0.0041, 0.0380)	Hap 0
			5244404	6	213	6	213	219	0.0274	(0.0079, 0.0343)	Unclassified
			5246356	22	457	28	670	698	0.0401	(0.0169, 0.0252)	Hap 0
			5248330	13	521	13	521	534	0.0243	(0.0156, 0.0265)	Unclassified
			5253166	10	336	23	857	880	0.0261	(0.0178, 0.0244)	Hap 0
	Mother	type α SNPs	5255843	322	234	322	234	556	0.5791	(0.4699, 0.5853)	Unclassified
		type β SNPs	5248200	261	259	261	259	520	0.5019	(0.4108, 0.5341)	Unclassified

			5250551	283	257	544	516	1060	0.5132	(0.4422, 0.5027)	Hap 0
			5258336	85	95	85	95	180	0.4722	(0.2943, 0.6506)	Unclassified
F09	Father	type α SNPs	5229835	708	46	708	46	754	0.9390	(0.9831, 0.9876)	Hap 1
			5237435	11	0	11	0	11	1.0000	(0.8302, 1.1405)	Unclassified
			5246514	889	84	900	84	984	0.9146	(0.9836, 0.9871)	Hap 1
		type β SNPs	5223750	3	951	3	951	954	0.0031	(0.0194, 0.0253)	Hap 1
			5223871	5	1154	5	1154	1159	0.0043	(0.0200, 0.0248)	Hap 1
			5224054	1	1027	1	1027	1028	0.0010	(0.0197, 0.0251)	Hap 1
			5224660	2	910	2	910	912	0.0022	(0.0193, 0.0254)	Hap 1
			5226579	0	116	0	116	116	0.0000	(-0.0017, 0.0464)	Unclassified
			5227471	0	754	0	870	870	0.0000	(0.0192, 0.0256)	Hap 1
			5227950	1	863	1	863	864	0.0012	(0.0191, 0.0256)	Hap 1
			5227975	0	827	0	827	827	0.0000	(0.0190, 0.0257)	Hap 1
			5229010	0	1158	0	1158	1158	0.0000	(0.0200, 0.0248)	Hap 1
			5229743	1	849	1	849	850	0.0012	(0.0191, 0.0257)	Hap 1
			5229745	0	847	0	847	847	0.0000	(0.0191, 0.0257)	Hap 1
			5230302	2	1144	2	1144	1146	0.0017	(0.0199, 0.0248)	Hap 1
			5231897	4	1150	4	1150	1154	0.0035	(0.0200, 0.0248)	Hap 1
			5232146	0	71	0	71	71	0.0000	(-0.0169, 0.0617)	Unclassified
			5232212	0	95	0	166	166	0.0000	(0.0056, 0.0392)	Hap 1
			5232573	1	983	1	983	984	0.0010	(0.0195, 0.0252)	Hap 1

			5233643	0	59	0	59	59	0.0000	(-0.0249, 0.0696)	Unclassified
			5233697	0	219	0	278	278	0.0000	(0.0123, 0.0324)	Hap 1
			5233836	2	709	2	709	711	0.0028	(0.0184, 0.0263)	Hap 1
			5234029	0	15	0	15	15	0.0000	(-0.1636, 0.2083)	Unclassified
			5234542	0	89	0	104	104	0.0000	(-0.0045, 0.0492)	Unclassified
			5234587	0	129	0	233	233	0.0000	(0.0104, 0.0343)	Hap 1
			5236417	2	1127	2	1127	1129	0.0018	(0.0199, 0.0248)	Hap 1
			5236740	2	536	2	536	538	0.0037	(0.0172, 0.0276)	Hap 1
			5237284	0	283	0	283	283	0.0000	(0.0125, 0.0322)	Hap 1
			5243757	0	855	0	855	855	0.0000	(0.0191, 0.0256)	Hap 1
			5244144	2	781	2	781	783	0.0026	(0.0188, 0.0259)	Hap 1
			5244299	0	566	0	566	566	0.0000	(0.0174, 0.0273)	Hap 1
			5244814	0	80	0	80	80	0.0000	(-0.0125, 0.0572)	Unclassified
			5245507	3	808	3	888	891	0.0034	(0.0192, 0.0255)	Hap 1
			5246000	4	946	4	946	950	0.0042	(0.0194, 0.0253)	Hap 1
			5246042	4	916	4	916	920	0.0043	(0.0193, 0.0254)	Hap 1
			5246203	9	1000	9	1000	1009	0.0089	(0.0196, 0.0251)	Hap 1
			5246512	4	957	4	957	961	0.0042	(0.0195, 0.0253)	Hap 1
			5247141	1	840	1	840	841	0.0012	(0.0191, 0.0257)	Hap 1
			5247733	0	1111	0	1111	1111	0.0000	(0.0199, 0.0249)	Hap 1
			5247791	1	1188	1	1188	1189	0.0008	(0.0200, 0.0247)	Hap 1
			5248243	4	920	4	920	924	0.0043	(0.0194, 0.0254)	Hap 1

			5248641	0	946	0	946	946	0.0000	(0.0194, 0.0253)	Hap 1
			5248852	1	418	1	418	419	0.0024	(0.0157, 0.0290)	Hap 1
			5249004	2	740	2	740	742	0.0027	(0.0186, 0.0261)	Hap 1
	Mather	type α SNPs	5257189	470	454	470	454	924	0.5087	(0.4983, 0.5619)	Unclassified
			5257324	496	507	966	961	1927	0.5013	(0.5148, 0.5453)	Hap 1
			5259000	549	387	549	387	936	0.5865	(0.4987, 0.5615)	Hap 0
			5262001	478	492	478	492	970	0.4928	(0.4998, 0.5604)	Hap 1
F10	Father	type α SNPs	5223750	459	3	459	3	462	0.9935	(0.9834, 0.9912)	Hap 0
			5223970	518	0	518	0	518	1.0000	(0.9838, 0.9908)	Hap 0
			5224660	467	2	467	2	469	0.9957	(0.9835, 0.9911)	Hap 0
			5227950	505	1	505	1	506	0.9980	(0.9838, 0.9908)	Hap 0
			5227975	499	0	499	0	499	1.0000	(0.9837, 0.9909)	Hap 0
			5228708	549	1	549	1	550	0.9982	(0.9840, 0.9905)	Hap 0
			5229196	427	0	427	0	427	1.0000	(0.9831, 0.9915)	Hap 0
			5229743	413	1	413	1	414	0.9976	(0.9830, 0.9916)	Hap 0
			5229745	411	1	411	1	412	0.9976	(0.9830, 0.9916)	Hap 0
			5231897	542	2	542	2	544	0.9963	(0.9840, 0.9906)	Hap 0
			5232146	44	0	44	0	44	1.0000	(0.9466, 1.0280)	Unclassified
			5232573	544	2	588	2	590	0.9966	(0.9843, 0.9903)	Hap 0
			5233094	215	0	215	0	215	1.0000	(0.9790, 0.9956)	Hap 0
			5233643	30	0	30	0	30	1.0000	(0.9277, 1.0469)	Unclassified

			5234587	69	0	99	0	99	1.0000	(0.9692, 1.0054)	Unclassified
			5236417	561	2	660	2	662	0.9970	(0.9846, 0.9900)	Hap 0
			5236851	574	0	574	0	574	1.0000	(0.9842, 0.9904)	Hap 0
			5243559	63	0	63	0	63	1.0000	(0.9589, 1.0157)	Unclassified
			5243613	170	0	233	0	233	1.0000	(0.9796, 0.9950)	Hap 0
			5243757	504	1	504	1	505	0.9980	(0.9838, 0.9908)	Hap 0
			5244299	380	3	380	3	383	0.9922	(0.9826, 0.9920)	Hap 0
			5244404	396	0	396	0	396	1.0000	(0.9828, 0.9918)	Hap 0
			5245507	430	0	430	0	430	1.0000	(0.9831, 0.9915)	Hap 0
			5246356	543	0	543	0	543	1.0000	(0.9840, 0.9906)	Hap 0
			5246512	418	0	418	0	418	1.0000	(0.9830, 0.9916)	Hap 0
			5247141	431	4	431	4	435	0.9908	(0.9832, 0.9914)	Unclassified
			5247791	453	2	884	6	890	0.9933	(0.9853, 0.9893)	Hap 0
			5248243	468	0	468	0	468	1.0000	(0.9835, 0.9911)	Hap 0
			5248641	445	4	445	4	449	0.9911	(0.9833, 0.9913)	Unclassified
			5248770	362	0	807	4	811	0.9951	(0.9851, 0.9895)	Hap 0
			5248852	243	1	243	1	244	0.9959	(0.9800, 0.9946)	Hap 0
			5249004	350	3	350	3	353	0.9915	(0.9822, 0.9924)	Unclassified
	Mather	type β SNPs	5268681	205	253	205	253	458	0.4476	(0.3979, 0.5520)	Unclassified
F11	Father	type α SNPs	5223750	590	0	590	0	590	1.0000	(0.9762, 0.9813)	Hap 0
			5224660	503	0	503	0	503	1.0000	(0.9757, 0.9818)	Hap 0

			5227950	545	0	545	0	545	1.0000	(0.9760, 0.9815)	Hap 0
			5227975	516	0	516	0	516	1.0000	(0.9758, 0.9817)	Hap 0
			5229509	621	0	621	0	621	1.0000	(0.9763, 0.9812)	Hap 0
			5229743	369	2	369	2	371	0.9946	(0.9746, 0.9829)	Hap 0
			5231897	560	4	560	4	564	0.9929	(0.9760, 0.9815)	Hap 0
			5232146	47	0	47	0	47	1.0000	(0.9463, 1.0112)	Unclassified
			5232573	553	4	600	4	604	0.9934	(0.9762, 0.9813)	Hap 0
			5233643	35	0	35	0	35	1.0000	(0.9352, 1.0223)	Unclassified
			5234587	70	0	105	0	105	1.0000	(0.9642, 0.9933)	Hap 0
			5236417	685	1	685	1	686	0.9985	(0.9765, 0.9810)	Hap 0
			5243757	520	4	520	4	524	0.9924	(0.9758, 0.9817)	Hap 0
			5244299	415	0	415	0	415	1.0000	(0.9751, 0.9824)	Hap 0
			5245507	477	0	477	0	477	1.0000	(0.9756, 0.9819)	Hap 0
			5246512	590	2	590	2	592	0.9966	(0.9762, 0.9813)	Hap 0
			5247141	563	3	563	3	566	0.9947	(0.9761, 0.9814)	Hap 0
			5247791	631	2	631	2	633	0.9968	(0.9763, 0.9812)	Hap 0
			5248243	542	1	542	1	543	0.9982	(0.9759, 0.9816)	Hap 0
			5248641	561	1	561	1	562	0.9982	(0.9760, 0.9815)	Hap 0
			5248770	410	2	410	2	412	0.9951	(0.9751, 0.9824)	Hap 0
			5250168	508	0	508	0	508	1.0000	(0.9758, 0.9818)	Hap 0
			5252251	352	0	352	0	352	1.0000	(0.9744, 0.9831)	Hap 0
			5253477	354	0	354	0	354	1.0000	(0.9744, 0.9831)	Hap 0

			5255912	461	0	461	0	461	1.0000	(0.9754, 0.9821)	Hap 0
			5256647	344	0	344	0	344	1.0000	(0.9743, 0.9832)	Hap 0
			5257778	629	0	629	0	629	1.0000	(0.9763, 0.9812)	Hap 0
			5258162	344	0	344	0	344	1.0000	(0.9743, 0.9832)	Hap 0
			5258265	71	0	71	0	71	1.0000	(0.9573, 1.0002)	Unclassified
			5258429	255	0	326	0	326	1.0000	(0.9741, 0.9834)	Hap 0
			5258490	371	0	371	0	371	1.0000	(0.9746, 0.9829)	Hap 0
			5258592	606	0	606	0	606	1.0000	(0.9762, 0.9813)	Hap 0
			5258827	596	1	596	1	597	0.9983	(0.9762, 0.9813)	Hap 0
			5258852	630	0	630	0	630	1.0000	(0.9763, 0.9812)	Hap 0
			5258856	622	3	622	3	625	0.9952	(0.9763, 0.9812)	Hap 0
			5258989	531	0	531	0	531	1.0000	(0.9759, 0.9816)	Hap 0
			5259289	316	0	316	0	316	1.0000	(0.9739, 0.9836)	Hap 0
			5259292	303	0	303	0	303	1.0000	(0.9737, 0.9838)	Hap 0
			5259419	51	0	51	0	51	1.0000	(0.9489, 1.0086)	Unclassified
			5259727	432	0	483	0	483	1.0000	(0.9756, 0.9819)	Hap 0
			5260458	568	0	568	0	568	1.0000	(0.9761, 0.9814)	Hap 0
			5260576	612	0	612	0	612	1.0000	(0.9763, 0.9812)	Hap 0
			5261239	639	0	639	0	639	1.0000	(0.9764, 0.9811)	Hap 0
			5263683	515	1	515	1	516	0.9981	(0.9758, 0.9817)	Hap 0
			5264146	692	0	692	0	692	1.0000	(0.9765, 0.9810)	Hap 0
			5265106	667	0	667	0	667	1.0000	(0.9765, 0.9810)	Hap 0

			5265680	299	0	299	0	299	1.0000	(0.9737, 0.9838)	Hap 0
			5266728	574	0	574	0	574	1.0000	(0.9761, 0.9814)	Hap 0
			5268406	592	0	592	0	592	1.0000	(0.9762, 0.9813)	Hap 0
			5268622	621	0	621	0	621	1.0000	(0.9763, 0.9812)	Hap 0
			5268797	583	0	583	0	583	1.0000	(0.9761, 0.9814)	Hap 0
			5269343	719	0	719	0	719	1.0000	(0.9766, 0.9809)	Hap 0
			5269806	365	1	365	1	366	0.9973	(0.9746, 0.9829)	Hap 0
			5269931	492	2	492	2	494	0.9960	(0.9757, 0.9818)	Hap 0
			5271671	680	0	680	0	680	1.0000	(0.9765, 0.9810)	Hap 0
			5272154	704	1	704	1	705	0.9986	(0.9766, 0.9809)	Hap 0
	Mather	type α SNPs	5223920	352	251	352	251	603	0.5837	(0.5173, 0.5784)	Hap 0
			5223970	378	243	378	243	621	0.6087	(0.5182, 0.5775)	Hap 0
			5228143	377	242	377	242	619	0.6090	(0.5181, 0.5776)	Hap 0
			5228708	383	252	383	252	635	0.6031	(0.5188, 0.5768)	Hap 0
			5229196	369	235	369	235	604	0.6109	(0.5173, 0.5783)	Hap 0
			5233447	11	4	11	4	15	0.7333	(-0.6803, 1.7759)	Unclassified
			5234781	80	51	91	55	146	0.6233	(0.4216, 0.6740)	Unclassified
			5236851	360	276	451	331	782	0.5767	(0.5243, 0.5714)	Hap 0
			5243559	36	35	36	35	71	0.5070	(0.2884, 0.8073)	Unclassified
			5243613	71	83	107	118	225	0.4756	(0.4659, 0.6297)	Unclassified
			5244404	272	164	379	282	661	0.5734	(0.5199, 0.5757)	Unclassified
			5246356	368	206	747	488	1235	0.6049	(0.5329,	Hap 0

										0.5627)	
			5248852	154	48	154	48	202	0.7624	(0.4566, 0.6390)	Hap 0
			5249004	300	171	300	171	471	0.6369	(0.5087, 0.5869)	Hap 0
F12	Father	type α SNPs	5248852	87	3	87	3	90	0.9667	(0.9678, 1.0087)	Hap 1
		type β SNPs	5250261	0	288	0	288	288	0.0000	(0.0074, 0.0293)	Hap 1
	Mather	type α SNPs	5223750	130	142	130	142	272	0.4779	(0.3783, 0.6668)	Unclassified
			5223871	148	150	278	292	570	0.4877	(0.4537, 0.5914)	Unclassified
			5224054	120	116	398	408	806	0.4938	(0.4739, 0.5712)	Unclassified
			5224660	129	148	527	556	1083	0.4866	(0.4863, 0.5588)	Unclassified
			5227471	110	116	637	672	1309	0.4866	(0.4926, 0.5525)	Hap 1
			5227950	44	63	44	63	107	0.4112	(0.1558, 0.8893)	Unclassified
			5227975	51	65	95	128	223	0.4260	(0.3466, 0.6985)	Unclassified
			5229010	175	170	270	298	568	0.4754	(0.4535, 0.5916)	Unclassified
			5229743	104	166	374	464	838	0.4463	(0.4757, 0.5694)	Hap 1
			5230302	220	232	220	232	452	0.4867	(0.4357, 0.6094)	Unclassified
			5231897	228	228	448	460	908	0.4934	(0.4793, 0.5658)	Unclassified
			5232146	19	24	467	484	951	0.4911	(0.4813, 0.5638)	Unclassified
			5232212	10	20	477	504	981	0.4862	(0.4826, 0.5626)	Unclassified
			5232573	152	123	629	627	1256	0.5008	(0.4913, 0.5538)	Unclassified
			5233643	100	95	729	722	1451	0.5024	(0.4955, 0.5496)	Unclassified
			5233697	143	139	872	861	1733	0.5032	(0.4999, 0.5452)	Unclassified

			5233836	181	181	1053	1042	2095	0.5026	(0.5038, 0.5413)	Hap 1
			5234029	163	177	163	177	340	0.4794	(0.4071, 0.6380)	Unclassified
			5234542	9	6	172	183	355	0.4845	(0.4120, 0.6331)	Unclassified
			5236417	187	239	359	422	781	0.4597	(0.4723, 0.5728)	Hap 1
			5236740	145	175	145	175	320	0.4531	(0.3999, 0.6452)	Unclassified
			5237284	89	76	234	251	485	0.4825	(0.4417, 0.6035)	Unclassified
			5243757	123	132	357	383	740	0.4824	(0.4695, 0.5756)	Unclassified
			5244144	47	56	404	439	843	0.4792	(0.4760, 0.5691)	Unclassified
			5244299	28	39	432	478	910	0.4747	(0.4794, 0.5657)	Hap 1
			5245507	114	145	114	145	259	0.4402	(0.3711, 0.6741)	Unclassified
			5246000	189	189	303	334	637	0.4757	(0.4610, 0.5842)	Unclassified
			5246203	226	225	529	559	1088	0.4862	(0.4865, 0.5586)	Hap 1
			5246512	168	180	168	180	348	0.4828	(0.4098, 0.6353)	Unclassified
			5247141	126	127	294	307	601	0.4892	(0.4573, 0.5878)	Unclassified
			5247733	138	182	432	489	921	0.4691	(0.4800, 0.5652)	Hap 1
			5247791	194	208	194	208	402	0.4826	(0.4249, 0.6202)	Unclassified
			5248243	211	186	405	394	799	0.5069	(0.4734, 0.5717)	Unclassified
			5248641	188	182	593	576	1169	0.5073	(0.4890, 0.5561)	Unclassified
			5248770	140	116	733	692	1425	0.5144	(0.4950, 0.5501)	Unclassified
			5248842	30	59	763	751	1514	0.5040	(0.4966, 0.5485)	Unclassified
			5257189	160	156	923	907	1830	0.5044	(0.5011, 0.5440)	Unclassified
			5259000	243	140	1166	1047	2213	0.5269	(0.5048, 0.5403)	Unclassified

F13	Father	type α SNPs	5223920	532	0	532	0	532	1.0000	(0.9830, 0.9896)	Hap 0
			5225021	129	0	129	0	129	1.0000	(0.9728, 0.9998)	Hap 0
			5228143	618	0	618	0	618	1.0000	(0.9835, 0.9891)	Hap 0
			5228708	628	0	628	0	628	1.0000	(0.9835, 0.9891)	Hap 0
			5229196	589	0	589	0	589	1.0000	(0.9834, 0.9893)	Hap 0
			5236792	559	0	559	0	559	1.0000	(0.9832, 0.9894)	Hap 0
			5236851	612	0	612	0	612	1.0000	(0.9835, 0.9892)	Hap 0
			5253586	571	0	571	0	571	1.0000	(0.9833, 0.9894)	Hap 0
	Mather	type α SNPs	5224660	317	237	317	237	554	0.5722	(0.4697, 0.5855)	Unclassified
			5227950	289	236	606	473	1079	0.5616	(0.4979, 0.5573)	Hap 0
			5227975	257	234	257	234	491	0.5234	(0.4623, 0.5929)	Unclassified
			5229743	187	220	444	454	898	0.4944	(0.4919, 0.5633)	Unclassified
			5231897	364	296	808	750	1558	0.5186	(0.5070, 0.5482)	Unclassified
			5232573	337	248	1145	998	2143	0.5343	(0.5126, 0.5425)	Unclassified
			5236417	294	283	1439	1281	2720	0.5290	(0.5158, 0.5394)	Unclassified
			5247876	301	244	1740	1525	3265	0.5329	(0.5178, 0.5374)	Unclassified
			5249004	260	172	2000	1697	3697	0.5410	(0.5189, 0.5363)	Hap 0
		type β SNPs	5249290	256	258	256	258	514	0.4981	(0.4100, 0.5348)	Unclassified

Supplemetary Table S7: Determining the paternally and maternally inherited haplotype by RHDO analysis of 20 kb amplicons.

Family			Genomic location	Counts for the allele on Hap 0	Counts for the allele on Hap 1	Cumulat-ive count for alleles on Hap 0	Cumulat-ive count for alleles on Hap 1	Total no. of reads	Fraction of total reads contributed by Hap 0 alleles	Classification threshold (upper, lower)	SPRT classification result
F01	Father	type α SNPs	5228143	1013	0	1013	0	1013	1.0000	(0.9837, 0.9870)	Hap 0
			5228708	993	3	993	3	996	0.9970	(0.9836, 0.9871)	Hap 0
			5229196	884	3	884	3	887	0.9966	(0.9834, 0.9873)	Hap 0
			5233447	24	0	24	0	24	1.0000	(0.9143, 1.0564)	Unclassified
			5234781	148	0	172	0	172	1.0000	(0.9754, 0.9953)	Hap 0
			5236851	947	2	947	2	949	0.9979	(0.9836, 0.9871)	Hap 0
			5243559	89	0	89	0	89	1.0000	(0.9662, 1.0045)	Unclassified
			5243613	211	0	300	0	300	1.0000	(0.9797, 0.9910)	Hap 0
			5244404	572	1	572	1	573	0.9983	(0.9824, 0.9883)	Hap 0
			5246356	822	1	822	1	823	0.9988	(0.9833, 0.9874)	Hap 0
		type β SNPs	5247992	45	990	45	990	1035	0.0435	(0.0197, 0.0251)	Hap 0
			5257189	47	882	47	882	929	0.0506	(0.0194, 0.0254)	Hap 0
			5259000	43	1010	43	1010	1053	0.0408	(0.0197, 0.0250)	Hap 0
	Mother	type α SNPs	5229835	342	300	342	300	642	0.5327	(0.4843, 0.5759)	Unclassified
			5237435	13	11	355	311	666	0.5330	(0.4859, 0.5742)	Unclassified
			5246514	471	331	826	642	1468	0.5627	(0.5100,	Hap 0

										0.5501)	
			5264411	531	395	531	395	926	0.5734	(0.4983, 0.5618)	Hap 0
		type β SNPs	5226579	40	67	40	67	107	0.3738	(0.1952, 0.7447)	Unclassified
			5227471	355	360	395	427	822	0.4805	(0.4342, 0.5057)	Unclassified
			5229010	482	459	877	886	1763	0.4974	(0.4533, 0.4866)	Hap 0
			5232212	35	48	35	48	83	0.4217	(0.1157, 0.8241)	Unclassified
			5233697	134	104	169	152	321	0.5265	(0.3783, 0.5615)	Unclassified
			5233836	364	335	533	487	1020	0.5225	(0.4411, 0.4988)	Hap 0
			5234029	9	10	9	10	19	0.4737	(-1.0774, 2.0173)	Unclassified
			5234542	25	37	34	47	81	0.4198	(0.1070, 0.8329)	Unclassified
			5236740	271	275	305	322	627	0.4864	(0.4230, 0.5168)	Unclassified
			5237284	117	145	422	467	889	0.4747	(0.4369, 0.5030)	Unclassified
			5244144	398	393	820	860	1680	0.4881	(0.4524, 0.4874)	Hap 0
			5245406	279	293	279	293	572	0.4878	(0.4185, 0.5213)	Unclassified
			5246000	408	368	687	661	1348	0.5096	(0.4481, 0.4917)	Hap 0
			5246203	394	324	394	324	718	0.5487	(0.4290, 0.5109)	Hap 0
			5247153	338	387	338	387	725	0.4662	(0.4294, 0.5105)	Unclassified
			5247733	439	464	777	851	1628	0.4773	(0.4519, 0.4880)	Unclassified
			5266843	452	493	1229	1344	2573	0.4777	(0.4585, 0.4814)	Unclassified
F02	Father	type α SNPs	5229835	897	4	897	4	901	0.9956	(0.9762, 0.9795)	Hap 0
			5237435	14	0	14	0	14	1.0000	(0.8703, 1.0854)	Unclassified
			5246514	1361	2	1375	2	1377	0.9985	(0.9767,	Hap 0

										0.9789)	
		type β SNPs	5226579	2	73	2	73	75	0.0267	(0.0019, 0.0629)	Unclassified
			5227471	97	842	99	915	1014	0.0976	(0.0302, 0.0347)	Hap 0
			5229010	152	1437	152	1437	1589	0.0957	(0.0310, 0.0339)	Hap 0
			5232212	3	88	3	88	91	0.0330	(0.0072, 0.0576)	Unclassified
			5233697	25	298	28	386	414	0.0676	(0.0269, 0.0379)	Hap 0
			5233836	126	944	126	944	1070	0.1178	(0.0303, 0.0346)	Hap 0
			5234029	1	22	1	22	23	0.0435	(-0.0672, 0.1320)	Unclassified
			5234542	3	61	4	83	87	0.0460	(0.0061, 0.0587)	Unclassified
			5236740	62	663	66	746	812	0.0813	(0.0296, 0.0352)	Hap 0
			5237284	29	299	29	299	328	0.0884	(0.0254, 0.0394)	Hap 0
			5244144	70	938	70	938	1008	0.0694	(0.0301, 0.0347)	Hap 0
			5245406	56	749	56	749	805	0.0696	(0.0296, 0.0353)	Hap 0
			5246000	138	1350	138	1350	1488	0.0927	(0.0309, 0.0340)	Hap 0
			5246203	139	1135	139	1135	1274	0.1091	(0.0306, 0.0342)	Hap 0
			5247153	94	962	94	962	1056	0.0890	(0.0302, 0.0346)	Hap 0
			5247733	165	1454	165	1454	1619	0.1019	(0.0310, 0.0338)	Hap 0
	Mother	type α SNPs	5228143	845	533	845	533	1378	0.6132	(0.5377, 0.5630)	Hap 0
			5228708	922	559	922	559	1481	0.6226	(0.5385, 0.5621)	Hap 0
			5229196	728	434	728	434	1162	0.6265	(0.5353, 0.5654)	Hap 0
			5233447	6	4	6	4	10	0.6000	(-1.1983, 2.2990)	Unclassified
			5234781	133	88	139	92	231	0.6017	(0.4746,	Unclassified

										0.6260)	
			5236851	822	558	961	650	1611	0.5965	(0.5395, 0.5612)	Hap 0
			5243559	67	45	67	45	112	0.5982	(0.3942, 0.7065)	Unclassified
			5243613	142	116	209	161	370	0.5649	(0.5031, 0.5976)	Unclassified
			5244383	464	264	673	425	1098	0.6129	(0.5344, 0.5663)	Hap 0
			5244404	427	237	427	237	664	0.6431	(0.5240, 0.5767)	Hap 0
			5246356	947	513	947	513	1460	0.6486	(0.5384, 0.5623)	Hap 0
			5248852	328	48	328	48	376	0.8723	(0.5038, 0.5968)	Hap 0
			5262368	557	414	557	414	971	0.5736	(0.5323, 0.5683)	Hap 0
		type β SNPs	5248200	726	777	726	777	1503	0.4830	(0.4380, 0.4613)	Hap 0
			5248770	517	331	517	331	848	0.6097	(0.4290, 0.4703)	Hap 0
			5248842	286	54	286	54	340	0.8412	(0.3982, 0.5011)	Hap 0
F03	Father	type β SNPs	5247992	86	616	86	616	702	0.1225	(0.0253, 0.0322)	Hap 0
			5257189	52	326	52	326	378	0.1376	(0.0223, 0.0352)	Hap 0
			5259000	41	399	41	399	440	0.0932	(0.0232, 0.0343)	Hap 0
	Mother	type α SNPs	5228708	235	148	235	148	383	0.6136	(0.4888, 0.5966)	Hap 0
			5229196	210	101	210	101	311	0.6752	(0.4763, 0.6091)	Hap 0
			5235864	32	22	32	22	54	0.5926	(0.1603, 0.9251)	Unclassified
			5236851	216	102	248	124	372	0.6667	(0.4872, 0.5982)	Hap 0
			5243613	34	13	34	13	47	0.7234	(0.1033, 0.9821)	Unclassified
			5244404	126	58	160	71	231	0.6926	(0.4533, 0.6321)	Hap 0

			5246356	232	98	232	98	330	0.7030	(0.4801, 0.6053)	Hap 0
			5253586	214	104	214	104	318	0.6730	(0.4778, 0.6076)	Hap 0
		type β SNPs	5227471	104	114	104	114	218	0.4771	(0.3626, 0.5520)	Unclassified
			5229010	215	230	319	344	663	0.4811	(0.4261, 0.4884)	Unclassified
			5233697	16	24	335	368	703	0.4765	(0.4279, 0.4867)	Unclassified
			5233836	122	154	457	522	979	0.4668	(0.4362, 0.4784)	Unclassified
			5234542	4	6	461	528	989	0.4661	(0.4364, 0.4782)	Unclassified
			5236740	96	89	557	617	1174	0.4744	(0.4397, 0.4749)	Unclassified
			5237284	38	18	595	635	1230	0.4837	(0.4405, 0.4741)	Hap 0
			5244144	102	111	102	111	213	0.4789	(0.3603, 0.5542)	Unclassified
			5245406	112	107	214	218	432	0.4954	(0.4095, 0.5051)	Unclassified
			5246000	209	213	423	431	854	0.4953	(0.4331, 0.4815)	Hap 0
			5246203	149	179	149	179	328	0.4543	(0.3943, 0.5203)	Unclassified
			5247153	143	136	292	315	607	0.4811	(0.4233, 0.4913)	Unclassified
			5247733	205	232	497	547	1044	0.4761	(0.4375, 0.4771)	Unclassified
F04	Father	type α SNPs	5255989	818	0	818	0	818	1.0000	(0.9823, 0.9864)	Hap 0
			5257778	837	0	837	0	837	1.0000	(0.9824, 0.9864)	Hap 0
			5263683	636	0	636	0	636	1.0000	(0.9818, 0.9870)	Hap 0
			5264146	903	2	903	2	905	0.9978	(0.9825, 0.9862)	Hap 0
			5265106	826	0	826	0	826	1.0000	(0.9824, 0.9864)	Hap 0
			5265680	406	0	406	0	406	1.0000	(0.9803, 0.9885)	Hap 0

			5269931	690	1	690	1	691	0.9986	(0.9820, 0.9868)	Hap 0
			5271671	867	4	867	4	871	0.9954	(0.9825, 0.9863)	Hap 0
		type β SNPs	5248200	42	800	42	800	842	0.0499	(0.0205, 0.0269)	Hap 0
			5248842	20	136	20	136	156	0.1282	(0.0064, 0.0410)	Hap 0
	Mother	type α SNPs	5227950	327	214	327	214	541	0.6044	(0.4825, 0.5827)	Hap 0
			5227975	326	214	326	214	540	0.6037	(0.4824, 0.5828)	Hap 0
			5228143	458	398	458	398	856	0.5350	(0.5009, 0.5643)	Unclassified
			5228708	457	377	915	775	1690	0.5414	(0.5165, 0.5486)	Unclassified
			5229196	481	336	1396	1111	2507	0.5568	(0.5218, 0.5434)	Hap 0
			5229743	409	156	409	156	565	0.7239	(0.4846, 0.5806)	Hap 0
			5229745	410	151	410	151	561	0.7308	(0.4843, 0.5809)	Hap 0
			5230302	504	434	504	434	938	0.5373	(0.5037, 0.5615)	Unclassified
			5231897	515	377	1019	811	1830	0.5568	(0.5178, 0.5474)	Hap 0
			5232146	37	28	37	28	65	0.5692	(0.1154, 0.9497)	Unclassified
			5232573	425	344	462	372	834	0.5540	(0.5001, 0.5651)	Unclassified
			5233447	12	10	474	382	856	0.5537	(0.5009, 0.5643)	Unclassified
			5233643	29	28	503	410	913	0.5509	(0.5029, 0.5623)	Unclassified
			5234587	61	28	564	438	1002	0.5629	(0.5055, 0.5597)	Hap 0
			5234781	102	77	102	77	179	0.5698	(0.3811, 0.6841)	Unclassified
			5236417	526	403	628	480	1108	0.5668	(0.5081, 0.5571)	Hap 0
			5236851	475	369	475	369	844	0.5628	(0.5005, 0.5647)	Unclassified

			5243559	48	44	523	413	936	0.5588	(0.5036, 0.5616)	Unclassified
			5243613	95	104	618	517	1135	0.5445	(0.5087, 0.5565)	Unclassified
			5243757	361	309	979	826	1805	0.5424	(0.5176, 0.5476)	Unclassified
			5244299	209	161	1188	987	2175	0.5462	(0.5201, 0.5451)	Hap 0
			5244404	284	179	284	179	463	0.6134	(0.4740, 0.5912)	Hap 0
			5245507	346	273	346	273	619	0.5590	(0.4888, 0.5764)	Unclassified
			5246042	416	323	762	596	1358	0.5611	(0.5126, 0.5526)	Hap 0
			5246356	532	342	532	342	874	0.6087	(0.5016, 0.5636)	Hap 0
			5246512	368	338	368	338	706	0.5212	(0.4942, 0.5710)	Unclassified
			5247141	362	280	730	618	1348	0.5415	(0.5125, 0.5527)	Unclassified
			5247791	512	480	1242	1098	2340	0.5308	(0.5210, 0.5442)	Unclassified
			5248243	403	376	1645	1474	3119	0.5274	(0.5239, 0.5413)	Unclassified
			5248641	416	347	2061	1821	3882	0.5309	(0.5256, 0.5396)	Unclassified
			5248770	257	205	2318	2026	4344	0.5336	(0.5264, 0.5388)	Unclassified
			5248852	170	96	2488	2122	4610	0.5397	(0.5267, 0.5385)	Hap 0
			5249715	351	154	351	154	505	0.6950	(0.4789, 0.5863)	Hap 0
			5257189	455	359	455	359	814	0.5590	(0.4993, 0.5659)	Unclassified
			5259000	499	305	954	664	1618	0.5896	(0.5158, 0.5494)	Hap 0
			5265941	436	324	436	324	760	0.5737	(0.4969, 0.5683)	Hap 0
		type β SNPs	5248329	400	431	400	431	831	0.4813	(0.4348, 0.5000)	Unclassified
			5249370	309	354	709	785	1494	0.4746	(0.4493, 0.4856)	Unclassified
			5256431	365	318	1074	1103	2177	0.4933	(0.4550,	Hap 0

										0.4799)	
			5259534	39	15	39	15	54	0.7222	(-0.0347, 0.9695)	Unclassified
			5262782	438	469	477	484	961	0.4964	(0.4392, 0.4956)	Hap 0
			5263577	390	408	390	408	798	0.4887	(0.4334, 0.5014)	Unclassified
			5264929	437	312	827	720	1547	0.5346	(0.4499, 0.4849)	Hap 0
			5265366	66	52	66	52	118	0.5593	(0.2376, 0.6972)	Unclassified
			5265475	8	4	74	56	130	0.5692	(0.2588, 0.6760)	Unclassified
			5266961	378	368	452	424	876	0.5160	(0.4365, 0.4984)	Hap 0
			5269140	593	512	593	512	1105	0.5367	(0.4429, 0.4919)	Hap 0
			5269799	292	295	292	295	587	0.4974	(0.4212, 0.5136)	Unclassified
			5270418	477	419	769	714	1483	0.5185	(0.4491, 0.4857)	Hap 0
F05	Father	type α SNPs	5255044	544	1	544	1	545	0.9982	(0.9769, 0.9825)	Hap 0
		type β SNPs	5248329	106	835	106	835	941	0.1126	(0.0274, 0.0325)	Hap 0
			5249004	73	709	73	709	782	0.0934	(0.0269, 0.0330)	Hap 0
	Mother	type α SNPs	5231897	518	523	518	523	1041	0.4976	(0.5265, 0.5640)	Hap 1
			5232212	38	17	38	17	55	0.6909	(0.1911, 0.8994)	Unclassified
			5232573	503	482	541	499	1040	0.5202	(0.5265, 0.5640)	Hap 1
			5233643	28	31	28	31	59	0.4746	(0.2151, 0.8754)	Unclassified
			5233697	120	131	148	162	310	0.4774	(0.4824, 0.6081)	Hap 1
			5234029	6	6	6	6	12	0.5000	(-1.0781, 2.1686)	Unclassified
			5234542	27	29	33	35	68	0.4853	(0.2588, 0.8317)	Unclassified

			5234587	45	41	78	76	154	0.5065	(0.4188, 0.6717)	Unclassified
			5243856	465	412	543	488	1031	0.5267	(0.5264, 0.5641)	Unclassified
			5257778	450	507	993	995	1988	0.4995	(0.5354, 0.5550)	Hap 1
			5263683	452	370	452	370	822	0.5499	(0.5215, 0.5689)	Unclassified
			5264146	511	471	963	841	1804	0.5338	(0.5344, 0.5560)	Hap 1
			5265106	511	531	511	531	1042	0.4904	(0.5266, 0.5639)	Hap 1
			5265680	254	253	254	253	507	0.5010	(0.5068, 0.5837)	Hap 1
			5269931	420	305	420	305	725	0.5793	(0.5184, 0.5721)	Hap 0
			5271671	563	472	563	472	1035	0.5440	(0.5264, 0.5641)	Unclassified
		type β SNPs	5247992	383	745	383	745	1128	0.3395	(0.4375, 0.4720)	Hap 1
			5250168	302	377	302	377	679	0.4448	(0.4261, 0.4834)	Unclassified
			5252251	222	423	524	800	1324	0.3958	(0.4400, 0.4695)	Hap 1
			5253462	180	404	180	404	584	0.3082	(0.4214, 0.4881)	Hap 1
			5253477	208	376	208	376	584	0.3562	(0.4214, 0.4881)	Hap 1
			5255912	288	421	288	421	709	0.4062	(0.4273, 0.4822)	Hap 1
			5256647	249	394	249	394	643	0.3872	(0.4245, 0.4850)	Hap 1
			5258038	307	485	307	485	792	0.3876	(0.4302, 0.4793)	Hap 1
			5258162	197	344	197	344	541	0.3641	(0.4187, 0.4908)	Hap 1
			5258265	33	79	33	79	112	0.2946	(0.2808, 0.6287)	Unclassified
			5258429	136	250	169	329	498	0.3394	(0.4156, 0.4939)	Hap 1
			5258490	199	450	199	450	649	0.3066	(0.4247, 0.4848)	Hap 1
			5258592	367	639	367	639	1006	0.3648	(0.4354,	Hap 1

										0.4741)	
			5258827	393	637	393	637	1030	0.3816	(0.4358, 0.4737)	Hap 1
			5258852	418	670	418	670	1088	0.3842	(0.4368, 0.4727)	Hap 1
			5258856	415	673	415	673	1088	0.3814	(0.4368, 0.4727)	Hap 1
			5258989	345	593	345	593	938	0.3678	(0.4340, 0.4755)	Hap 1
			5259103	352	666	352	666	1018	0.3458	(0.4356, 0.4739)	Hap 1
			5259289	157	281	157	281	438	0.3584	(0.4103, 0.4992)	Hap 1
			5259292	155	276	155	276	431	0.3596	(0.4096, 0.5000)	Hap 1
			5259419	22	40	22	40	62	0.3548	(0.1406, 0.7689)	Unclassified
			5259727	320	405	342	445	787	0.4346	(0.4300, 0.4795)	Unclassified
			5260458	363	604	705	1049	1754	0.4019	(0.4436, 0.4659)	Hap 1
			5260576	364	559	364	559	923	0.3944	(0.4336, 0.4759)	Hap 1
			5261239	413	601	413	601	1014	0.4073	(0.4355, 0.4740)	Hap 1
			5266728	395	608	395	608	1003	0.3938	(0.4353, 0.4742)	Hap 1
			5268406	377	593	377	593	970	0.3887	(0.4347, 0.4748)	Hap 1
			5268622	398	623	398	623	1021	0.3898	(0.4357, 0.4738)	Hap 1
			5268797	430	607	430	607	1037	0.4147	(0.4360, 0.4735)	Hap 1
			5269343	460	734	460	734	1194	0.3853	(0.4384, 0.4711)	Hap 1
			5269806	264	407	264	407	671	0.3934	(0.4257, 0.4838)	Hap 1
			5270246	399	405	399	405	804	0.4963	(0.4305, 0.4790)	Hap 0
			5270343	380	177	380	177	557	0.6822	(0.4198, 0.4897)	Hap 0
			5272154	461	742	461	742	1203	0.3832	(0.4386, 0.4709)	Hap 1

F06	Father	type α SNPs	5229835	147	0	147	0	147	1.0000	(0.9700, 0.9912)	Hap 0
			5237435	25	0	25	0	25	1.0000	(0.9180, 1.0432)	Unclassified
			5246514	418	0	443	0	443	1.0000	(0.9771, 0.9841)	Hap 0
		type β SNPs	5226579	0	11	0	11	11	0.0000	(-0.1922, 0.2497)	Unclassified
			5227950	3	135	3	146	149	0.0201	(0.0124, 0.0451)	Unclassified
			5227975	5	148	8	294	302	0.0265	(0.0207, 0.0368)	Unclassified
			5229010	29	348	37	642	679	0.0545	(0.0252, 0.0323)	Hap 0
			5229743	11	244	11	244	255	0.0431	(0.0192, 0.0383)	Hap 0
			5229745	10	248	10	248	258	0.0388	(0.0193, 0.0382)	Hap 0
			5230302	23	442	23	442	465	0.0495	(0.0235, 0.0340)	Hap 0
			5231897	29	471	29	471	500	0.0580	(0.0239, 0.0336)	Hap 0
			5232146	2	67	2	67	69	0.0290	(-0.0065, 0.0640)	Unclassified
			5232212	3	72	5	139	144	0.0347	(0.0119, 0.0456)	Unclassified
			5232573	27	322	32	461	493	0.0649	(0.0238, 0.0337)	Hap 0
			5233643	13	209	13	209	222	0.0586	(0.0178, 0.0397)	Hap 0
			5233697	11	314	11	314	325	0.0338	(0.0213, 0.0362)	Unclassified
			5233836	32	398	43	712	755	0.0570	(0.0255, 0.0320)	Hap 0
			5234029	28	421	28	421	449	0.0624	(0.0233, 0.0342)	Hap 0
			5234542	0	21	0	21	21	0.0000	(-0.0870, 0.1445)	Unclassified
			5234587	0	13	0	34	34	0.0000	(-0.0427, 0.1002)	Unclassified
			5236417	24	424	24	458	482	0.0498	(0.0237, 0.0338)	Hap 0

			5236740	29	381	29	381	410	0.0707	(0.0228, 0.0347)	Hap 0
			5237284	6	159	6	159	165	0.0364	(0.0140, 0.0435)	Unclassified
			5237498	0	14	6	173	179	0.0335	(0.0152, 0.0423)	Unclassified
			5243757	14	209	20	382	402	0.0498	(0.0227, 0.0348)	Hap 0
			5244144	6	125	6	125	131	0.0458	(0.0102, 0.0473)	Unclassified
			5244299	9	81	15	206	221	0.0679	(0.0178, 0.0397)	Hap 0
			5245406	9	141	9	141	150	0.0600	(0.0125, 0.0449)	Hap 0
			5245507	12	140	12	140	152	0.0789	(0.0128, 0.0447)	Hap 0
			5246000	30	303	30	303	333	0.0901	(0.0214, 0.0360)	Hap 0
			5246042	26	334	26	334	360	0.0722	(0.0220, 0.0355)	Hap 0
			5246203	52	408	52	408	460	0.1130	(0.0235, 0.0340)	Hap 0
			5246512	35	388	35	388	423	0.0827	(0.0230, 0.0345)	Hap 0
			5247141	21	242	21	242	263	0.0798	(0.0195, 0.0380)	Hap 0
			5247153	21	226	21	226	247	0.0850	(0.0189, 0.0386)	Hap 0
			5247733	28	338	28	338	366	0.0765	(0.0221, 0.0354)	Hap 0
			5247791	27	459	27	459	486	0.0556	(0.0237, 0.0337)	Hap 0
			5248243	35	406	35	406	441	0.0794	(0.0232, 0.0343)	Hap 0
			5248641	20	286	20	286	306	0.0654	(0.0208, 0.0367)	Hap 0
	Mother	type α SNPs	5230648	94	111	94	111	205	0.4585	(0.4421, 0.6434)	Unclassified
		type β SNPs	5248200	262	205	262	205	467	0.5610	(0.4131, 0.5015)	Hap 0
			5248842	67	58	67	58	125	0.5360	(0.2922, 0.6224)	Unclassified

F07	Father	type α SNPs	5234678	114	0	114	0	114	1.0000	(0.9690, 0.9978)	Hap 0
			5250168	395	0	395	0	395	1.0000	(0.9793, 0.9876)	Hap 0
			5252251	322	1	322	1	323	0.9969	(0.9783, 0.9885)	Hap 0
			5253477	277	0	277	0	277	1.0000	(0.9775, 0.9893)	Hap 0
			5255912	408	0	408	0	408	1.0000	(0.9794, 0.9874)	Hap 0
			5256431	542	0	542	0	542	1.0000	(0.9804, 0.9865)	Hap 0
			5256647	264	0	264	0	264	1.0000	(0.9772, 0.9896)	Hap 0
			5258038	463	0	463	0	463	1.0000	(0.9799, 0.9870)	Hap 0
			5258162	356	0	356	0	356	1.0000	(0.9788, 0.9880)	Hap 0
			5258265	75	0	75	0	75	1.0000	(0.9615, 1.0053)	Unclassified
			5258429	228	0	303	0	303	1.0000	(0.9780, 0.9888)	Hap 0
			5258490	302	0	302	0	302	1.0000	(0.9780, 0.9889)	Hap 0
			5258592	503	0	503	0	503	1.0000	(0.9802, 0.9867)	Hap 0
			5258827	543	0	543	0	543	1.0000	(0.9804, 0.9864)	Hap 0
			5258852	558	0	558	0	558	1.0000	(0.9805, 0.9864)	Hap 0
			5258856	556	0	556	0	556	1.0000	(0.9805, 0.9864)	Hap 0
			5258989	478	0	478	0	478	1.0000	(0.9800, 0.9869)	Hap 0
			5259289	227	0	227	0	227	1.0000	(0.9762, 0.9907)	Hap 0
			5259292	212	0	212	0	212	1.0000	(0.9757, 0.9912)	Hap 0
			5259419	41	0	41	0	41	1.0000	(0.9434, 1.0234)	Unclassified
			5259534	41	0	82	0	82	1.0000	(0.9634, 1.0034)	Unclassified
			5259727	374	0	456	0	456	1.0000	(0.9798,	Hap 0

										0.9870)	
			5260458	484	0	484	0	484	1.0000	(0.9800, 0.9868)	Hap 0
			5260576	537	0	537	0	537	1.0000	(0.9804, 0.9865)	Hap 0
			5261239	505	0	505	0	505	1.0000	(0.9802, 0.9867)	Hap 0
			5262782	628	0	628	0	628	1.0000	(0.9808, 0.9860)	Hap 0
			5263577	575	0	575	0	575	1.0000	(0.9806, 0.9863)	Hap 0
			5264929	401	0	401	0	401	1.0000	(0.9793, 0.9875)	Hap 0
			5265366	67	1	67	1	68	0.9853	(0.9593, 1.0076)	Unclassified
			5265475	11	0	78	1	79	0.9873	(0.9627, 1.0042)	Unclassified
			5266728	490	0	568	1	569	0.9982	(0.9805, 0.9863)	Hap 0
			5266961	444	0	444	0	444	1.0000	(0.9797, 0.9871)	Hap 0
			5268406	487	0	487	0	487	1.0000	(0.9801, 0.9868)	Hap 0
			5268622	613	0	613	0	613	1.0000	(0.9807, 0.9861)	Hap 0
			5268797	509	0	509	0	509	1.0000	(0.9802, 0.9866)	Hap 0
			5269140	616	1	616	1	617	0.9984	(0.9808, 0.9861)	Hap 0
			5269343	502	0	502	0	502	1.0000	(0.9802, 0.9867)	Hap 0
			5269799	345	0	345	0	345	1.0000	(0.9787, 0.9882)	Hap 0
			5269806	356	2	356	2	358	0.9944	(0.9788, 0.9880)	Hap 0
			5270418	525	0	525	0	525	1.0000	(0.9803, 0.9865)	Hap 0
			5270539	408	0	408	0	408	1.0000	(0.9794, 0.9874)	Hap 0
			5272154	593	0	593	0	593	1.0000	(0.9807, 0.9862)	Hap 0
		type β SNPs	5246514	24	462	24	462	486	0.0494	(0.0196, 0.0304)	Hap 0

			5247904	28	531	28	531	559	0.0501	(0.0203, 0.0297)	Hap 0
			5250551	27	553	27	553	580	0.0466	(0.0205, 0.0295)	Hap 0
			5253586	19	445	19	445	464	0.0409	(0.0193, 0.0306)	Hap 0
			5258336	2	55	2	55	57	0.0351	(-0.0210, 0.0709)	Unclassified
	Mother	type α SNPs	5248770	165	177	165	177	342	0.4825	(0.4616, 0.6086)	Unclassified
			5249004	266	187	431	364	795	0.5421	(0.5035, 0.5668)	Unclassified
		type β SNPs	5247992	236	328	236	328	564	0.4184	(0.4203, 0.5094)	Hap 1
			5257575	203	299	203	299	502	0.4044	(0.4148, 0.5149)	Hap 1
F08	Father	type α SNPs	5226579	31	0	31	0	31	1.0000	(0.9300, 1.0426)	Unclassified
			5227471	331	0	362	0	362	1.0000	(0.9815, 0.9911)	Hap 0
			5229010	480	0	480	0	480	1.0000	(0.9827, 0.9899)	Hap 0
			5232212	97	0	97	0	97	1.0000	(0.9683, 1.0043)	Unclassified
			5233697	348	0	445	0	445	1.0000	(0.9824, 0.9902)	Hap 0
			5233836	443	0	443	0	443	1.0000	(0.9824, 0.9903)	Hap 0
			5234029	470	0	470	0	470	1.0000	(0.9826, 0.9900)	Hap 0
			5234542	23	0	23	0	23	1.0000	(0.9105, 1.0622)	Unclassified
			5236740	458	0	481	0	481	1.0000	(0.9827, 0.9899)	Hap 0
			5237284	190	0	190	0	190	1.0000	(0.9771, 0.9955)	Hap 0
			5237498	25	0	25	0	25	1.0000	(0.9165, 1.0561)	Unclassified
			5244144	168	0	193	0	193	1.0000	(0.9773, 0.9954)	Hap 0
			5245406	341	0	341	0	341	1.0000	(0.9812,	Hap 0

										0.9914)	
			5246000	449	0	449	0	449	1.0000	(0.9824, 0.9902)	Hap 0
			5246203	522	0	522	0	522	1.0000	(0.9830, 0.9897)	Hap 0
			5247733	435	0	435	0	435	1.0000	(0.9823, 0.9903)	Hap 0
		type β SNPs	5228143	27	507	27	507	534	0.0506	(0.0156, 0.0265)	Hap 0
			5228708	20	428	20	428	448	0.0446	(0.0146, 0.0275)	Hap 0
			5229196	30	371	30	371	401	0.0748	(0.0138, 0.0283)	Hap 0
			5233447	8	271	8	271	279	0.0287	(0.0107, 0.0314)	Unclassified
			5234781	1	20	9	291	300	0.0300	(0.0114, 0.0307)	Unclassified
			5236851	13	481	22	772	794	0.0277	(0.0174, 0.0247)	Hap 0
			5243559	3	52	3	52	55	0.0545	(-0.0315, 0.0737)	Unclassified
			5243613	9	107	12	159	171	0.0702	(0.0041, 0.0380)	Hap 0
			5244404	6	213	6	213	219	0.0274	(0.0079, 0.0343)	Unclassified
			5246356	22	457	28	670	698	0.0401	(0.0169, 0.0252)	Hap 0
			5248330	13	521	13	521	534	0.0243	(0.0156, 0.0265)	Unclassified
			5253166	10	336	23	857	880	0.0261	(0.0178, 0.0244)	Hap 0
	Mother	type α SNPs	5255843	322	234	322	234	556	0.5791	(0.4699, 0.5853)	Unclassified
		type β SNPs	5248200	261	259	261	259	520	0.5019	(0.4108, 0.5341)	Unclassified
			5250551	283	257	544	516	1060	0.5132	(0.4422, 0.5027)	Hap 0
			5258336	85	95	85	95	180	0.4722	(0.2943, 0.6506)	Unclassified
F09	Father	type α	5229835	708	46	708	46	754	0.9390	(0.9831, 0.9876)	Hap 1

		SNPs									
			5237435	11	0	11	0	11	1.0000	(0.8302, 1.1405)	Unclassified
			5246514	889	84	900	84	984	0.9146	(0.9836, 0.9871)	Hap 1
		type β SNPs	5226579	0	116	0	116	116	0.0000	(-0.0017, 0.0464)	Unclassified
			5227471	0	754	0	870	870	0.0000	(0.0192, 0.0256)	Hap 1
			5227950	1	863	1	863	864	0.0012	(0.0191, 0.0256)	Hap 1
			5227975	0	827	0	827	827	0.0000	(0.0190, 0.0257)	Hap 1
			5229010	0	1158	0	1158	1158	0.0000	(0.0200, 0.0248)	Hap 1
			5229743	1	849	1	849	850	0.0012	(0.0191, 0.0257)	Hap 1
			5229745	0	847	0	847	847	0.0000	(0.0191, 0.0257)	Hap 1
			5230302	2	1144	2	1144	1146	0.0017	(0.0199, 0.0248)	Hap 1
			5231897	4	1150	4	1150	1154	0.0035	(0.0200, 0.0248)	Hap 1
			5232146	0	71	0	71	71	0.0000	(-0.0169, 0.0617)	Unclassified
			5232212	0	95	0	166	166	0.0000	(0.0056, 0.0392)	Hap 1
			5232573	1	983	1	983	984	0.0010	(0.0195, 0.0252)	Hap 1
			5233643	0	59	0	59	59	0.0000	(-0.0249, 0.0696)	Unclassified
			5233697	0	219	0	278	278	0.0000	(0.0123, 0.0324)	Hap 1
			5233836	2	709	2	709	711	0.0028	(0.0184, 0.0263)	Hap 1
			5234029	0	15	0	15	15	0.0000	(-0.1636, 0.2083)	Unclassified
			5234542	0	89	0	104	104	0.0000	(-0.0045, 0.0492)	Unclassified
			5234587	0	129	0	233	233	0.0000	(0.0104, 0.0343)	Hap 1
			5236417	2	1127	2	1127	1129	0.0018	(0.0199, 0.0248)	Hap 1

			5236740	2	536	2	536	538	0.0037	(0.0172, 0.0276)	Hap 1
			5237284	0	283	0	283	283	0.0000	(0.0125, 0.0322)	Hap 1
			5243757	0	855	0	855	855	0.0000	(0.0191, 0.0256)	Hap 1
			5244144	2	781	2	781	783	0.0026	(0.0188, 0.0259)	Hap 1
			5244299	0	566	0	566	566	0.0000	(0.0174, 0.0273)	Hap 1
			5244814	0	80	0	80	80	0.0000	(-0.0125, 0.0572)	Unclassified
			5245507	3	808	3	888	891	0.0034	(0.0192, 0.0255)	Hap 1
			5246000	4	946	4	946	950	0.0042	(0.0194, 0.0253)	Hap 1
			5246042	4	916	4	916	920	0.0043	(0.0193, 0.0254)	Hap 1
			5246203	9	1000	9	1000	1009	0.0089	(0.0196, 0.0251)	Hap 1
			5246512	4	957	4	957	961	0.0042	(0.0195, 0.0253)	Hap 1
			5247141	1	840	1	840	841	0.0012	(0.0191, 0.0257)	Hap 1
			5247733	0	1111	0	1111	1111	0.0000	(0.0199, 0.0249)	Hap 1
			5247791	1	1188	1	1188	1189	0.0008	(0.0200, 0.0247)	Hap 1
			5248243	4	920	4	920	924	0.0043	(0.0194, 0.0254)	Hap 1
			5248641	0	946	0	946	946	0.0000	(0.0194, 0.0253)	Hap 1
			5248852	1	418	1	418	419	0.0024	(0.0157, 0.0290)	Hap 1
			5249004	2	740	2	740	742	0.0027	(0.0186, 0.0261)	Hap 1
			5260098	2	817	2	817	819	0.0024	(0.0190, 0.0258)	Hap 1
	Mather	type α SNPs	5257189	470	454	470	454	924	0.5087	(0.4983, 0.5619)	Unclassified
			5257324	496	507	966	961	1927	0.5013	(0.5148, 0.5453)	Hap 1
			5259000	549	387	549	387	936	0.5865	(0.4987,	Hap 0

										0.5615)	
			5262001	478	492	478	492	970	0.4928	(0.4998, 0.5604)	Hap 1
F10	Father	type α SNPs	5227950	505	1	505	1	506	0.9980	(0.9838, 0.9908)	Hap 0
			5227975	499	0	499	0	499	1.0000	(0.9837, 0.9909)	Hap 0
			5228708	549	1	549	1	550	0.9982	(0.9840, 0.9905)	Hap 0
			5229196	427	0	427	0	427	1.0000	(0.9831, 0.9915)	Hap 0
			5229743	413	1	413	1	414	0.9976	(0.9830, 0.9916)	Hap 0
			5229745	411	1	411	1	412	0.9976	(0.9830, 0.9916)	Hap 0
			5230302	538	0	538	0	538	1.0000	(0.9840, 0.9906)	Hap 0
			5231897	542	2	542	2	544	0.9963	(0.9840, 0.9906)	Hap 0
			5232146	44	0	44	0	44	1.0000	(0.9466, 1.0280)	Unclassified
			5232573	544	2	588	2	590	0.9966	(0.9843, 0.9903)	Hap 0
			5233094	215	0	215	0	215	1.0000	(0.9790, 0.9956)	Hap 0
			5233643	30	0	30	0	30	1.0000	(0.9277, 1.0469)	Unclassified
			5234580	62	0	92	0	92	1.0000	(0.9679, 1.0067)	Unclassified
			5234587	69	0	161	0	161	1.0000	(0.9762, 0.9984)	Hap 0
			5236417	561	2	561	2	563	0.9964	(0.9841, 0.9905)	Hap 0
			5236851	574	0	574	0	574	1.0000	(0.9842, 0.9904)	Hap 0
			5243559	63	0	63	0	63	1.0000	(0.9589, 1.0157)	Unclassified
			5243613	170	0	233	0	233	1.0000	(0.9796, 0.9950)	Hap 0
			5243757	504	1	504	1	505	0.9980	(0.9838, 0.9908)	Hap 0
			5244299	380	3	380	3	383	0.9922	(0.9826, 0.9920)	Hap 0

			5244404	396	0	396	0	396	1.0000	(0.9828, 0.9918)	Hap 0
			5245507	430	0	430	0	430	1.0000	(0.9831, 0.9915)	Hap 0
			5246042	418	4	418	4	422	0.9905	(0.9831, 0.9915)	Unclassified
			5246356	543	0	961	4	965	0.9959	(0.9854, 0.9892)	Hap 0
			5246512	418	0	418	0	418	1.0000	(0.9830, 0.9916)	Hap 0
			5247141	431	4	431	4	435	0.9908	(0.9832, 0.9914)	Unclassified
			5247791	453	2	884	6	890	0.9933	(0.9853, 0.9893)	Hap 0
			5248243	468	0	468	0	468	1.0000	(0.9835, 0.9911)	Hap 0
			5248641	445	4	445	4	449	0.9911	(0.9833, 0.9913)	Unclassified
			5248770	362	0	807	4	811	0.9951	(0.9851, 0.9895)	Hap 0
			5248852	243	1	243	1	244	0.9959	(0.9800, 0.9946)	Hap 0
			5249004	350	3	350	3	353	0.9915	(0.9822, 0.9924)	Unclassified
	Mother	type β SNPs	5268681	205	253	205	253	458	0.4476	(0.3979, 0.5520)	Unclassified
F11	Father	type α SNPs	5227950	545	0	545	0	545	1.0000	(0.9760, 0.9815)	Hap 0
			5227975	516	0	516	0	516	1.0000	(0.9758, 0.9817)	Hap 0
			5229509	621	0	621	0	621	1.0000	(0.9763, 0.9812)	Hap 0
			5229743	369	2	369	2	371	0.9946	(0.9746, 0.9829)	Hap 0
			5231897	560	4	560	4	564	0.9929	(0.9760, 0.9815)	Hap 0
			5232146	47	0	47	0	47	1.0000	(0.9463, 1.0112)	Unclassified
			5232573	553	4	600	4	604	0.9934	(0.9762, 0.9813)	Hap 0
			5233643	35	0	35	0	35	1.0000	(0.9352, 1.0223)	Unclassified

			5234587	70	0	105	0	105	1.0000	(0.9642, 0.9933)	Hap 0
			5236417	685	1	685	1	686	0.9985	(0.9765, 0.9810)	Hap 0
			5243757	520	4	520	4	524	0.9924	(0.9758, 0.9817)	Hap 0
			5244299	415	0	415	0	415	1.0000	(0.9751, 0.9824)	Hap 0
			5245507	477	0	477	0	477	1.0000	(0.9756, 0.9819)	Hap 0
			5246042	533	0	533	0	533	1.0000	(0.9759, 0.9816)	Hap 0
			5246512	590	2	590	2	592	0.9966	(0.9762, 0.9813)	Hap 0
			5247141	563	3	563	3	566	0.9947	(0.9761, 0.9814)	Hap 0
			5247791	631	2	631	2	633	0.9968	(0.9763, 0.9812)	Hap 0
			5248243	542	1	542	1	543	0.9982	(0.9759, 0.9816)	Hap 0
			5248641	561	1	561	1	562	0.9982	(0.9760, 0.9815)	Hap 0
			5248770	410	2	410	2	412	0.9951	(0.9751, 0.9824)	Hap 0
			5250168	508	0	508	0	508	1.0000	(0.9758, 0.9818)	Hap 0
			5252251	352	0	352	0	352	1.0000	(0.9744, 0.9831)	Hap 0
			5253477	354	0	354	0	354	1.0000	(0.9744, 0.9831)	Hap 0
			5255912	461	0	461	0	461	1.0000	(0.9754, 0.9821)	Hap 0
			5256647	344	0	344	0	344	1.0000	(0.9743, 0.9832)	Hap 0
			5257778	629	0	629	0	629	1.0000	(0.9763, 0.9812)	Hap 0
			5258162	344	0	344	0	344	1.0000	(0.9743, 0.9832)	Hap 0
			5258265	71	0	71	0	71	1.0000	(0.9573, 1.0002)	Unclassified
			5258429	255	0	326	0	326	1.0000	(0.9741, 0.9834)	Hap 0
			5258490	371	0	371	0	371	1.0000	(0.9746, 0.9829)	Hap 0

			5258592	606	0	606	0	606	1.0000	(0.9762, 0.9813)	Hap 0
			5258827	596	1	596	1	597	0.9983	(0.9762, 0.9813)	Hap 0
			5258852	630	0	630	0	630	1.0000	(0.9763, 0.9812)	Hap 0
			5258856	622	3	622	3	625	0.9952	(0.9763, 0.9812)	Hap 0
			5258989	531	0	531	0	531	1.0000	(0.9759, 0.9816)	Hap 0
			5259289	316	0	316	0	316	1.0000	(0.9739, 0.9836)	Hap 0
			5259292	303	0	303	0	303	1.0000	(0.9737, 0.9838)	Hap 0
			5259419	51	0	51	0	51	1.0000	(0.9489, 1.0086)	Unclassified
			5259727	432	0	483	0	483	1.0000	(0.9756, 0.9819)	Hap 0
			5260458	568	0	568	0	568	1.0000	(0.9761, 0.9814)	Hap 0
			5260576	612	0	612	0	612	1.0000	(0.9763, 0.9812)	Hap 0
			5261239	639	0	639	0	639	1.0000	(0.9764, 0.9811)	Hap 0
			5263683	515	1	515	1	516	0.9981	(0.9758, 0.9817)	Hap 0
			5264146	692	0	692	0	692	1.0000	(0.9765, 0.9810)	Hap 0
			5265106	667	0	667	0	667	1.0000	(0.9765, 0.9810)	Hap 0
			5265680	299	0	299	0	299	1.0000	(0.9737, 0.9838)	Hap 0
			5266728	574	0	574	0	574	1.0000	(0.9761, 0.9814)	Hap 0
			5268406	592	0	592	0	592	1.0000	(0.9762, 0.9813)	Hap 0
			5268622	621	0	621	0	621	1.0000	(0.9763, 0.9812)	Hap 0
			5268797	583	0	583	0	583	1.0000	(0.9761, 0.9814)	Hap 0
			5269343	719	0	719	0	719	1.0000	(0.9766, 0.9809)	Hap 0
			5269806	365	1	365	1	366	0.9973	(0.9746, 0.9829)	Hap 0

			5269931	492	2	492	2	494	0.9960	(0.9757, 0.9818)	Hap 0
			5271671	680	0	680	0	680	1.0000	(0.9765, 0.9810)	Hap 0
			5272154	704	1	704	1	705	0.9986	(0.9766, 0.9809)	Hap 0
	Mother	type α SNPs	5228143	377	242	377	242	619	0.6090	(0.5181, 0.5776)	Hap 0
			5228708	383	252	383	252	635	0.6031	(0.5188, 0.5768)	Hap 0
			5229196	369	235	369	235	604	0.6109	(0.5173, 0.5783)	Hap 0
			5233447	11	4	11	4	15	0.7333	(-0.6803, 1.7759)	Unclassified
			5234781	80	51	91	55	146	0.6233	(0.4216, 0.6740)	Unclassified
			5236851	360	276	451	331	782	0.5767	(0.5243, 0.5714)	Hap 0
			5243559	36	35	36	35	71	0.5070	(0.2884, 0.8073)	Unclassified
			5243613	71	83	107	118	225	0.4756	(0.4659, 0.6297)	Unclassified
			5244404	272	164	379	282	661	0.5734	(0.5199, 0.5757)	Unclassified
			5246356	368	206	747	488	1235	0.6049	(0.5329, 0.5627)	Hap 0
			5248852	154	48	154	48	202	0.7624	(0.4566, 0.6390)	Hap 0
			5249004	300	171	300	171	471	0.6369	(0.5087, 0.5869)	Hap 0
			5258042	323	217	323	217	540	0.5981	(0.5137, 0.5819)	Hap 0
			5260382	464	277	464	277	741	0.6262	(0.5230, 0.5727)	Hap 0
			5269235	434	310	434	310	744	0.5833	(0.5231, 0.5726)	Hap 0
F12	Father	type α SNPs	5248852	87	3	87	3	90	0.9667	(0.9678, 1.0087)	Hap 1
		type β SNPs	5250261	0	288	0	288	288	0.0000	(0.0074, 0.0293)	Hap 1
	Mother	type	5227471	110	116	110	116	226	0.4867	(0.3489,	Unclassified

		α SNPs								0.6962)	
			5227950	44	63	154	179	333	0.4625	(0.4047, 0.6404)	Unclassified
			5227975	51	65	205	244	449	0.4566	(0.4352, 0.6099)	Unclassified
			5229010	175	170	380	414	794	0.4786	(0.4731, 0.5720)	Unclassified
			5229743	104	166	484	580	1064	0.4549	(0.4857, 0.5594)	Hap 1
			5230302	220	232	220	232	452	0.4867	(0.4357, 0.6094)	Unclassified
			5231897	228	228	448	460	908	0.4934	(0.4793, 0.5658)	Unclassified
			5232146	19	24	467	484	951	0.4911	(0.4813, 0.5638)	Unclassified
			5232212	10	20	477	504	981	0.4862	(0.4826, 0.5626)	Unclassified
			5232573	152	123	629	627	1256	0.5008	(0.4913, 0.5538)	Unclassified
			5233643	100	95	729	722	1451	0.5024	(0.4955, 0.5496)	Unclassified
			5233697	143	139	872	861	1733	0.5032	(0.4999, 0.5452)	Unclassified
			5233836	181	181	1053	1042	2095	0.5026	(0.5038, 0.5413)	Hap 1
			5234029	163	177	163	177	340	0.4794	(0.4071, 0.6380)	Unclassified
			5234542	9	6	172	183	355	0.4845	(0.4120, 0.6331)	Unclassified
			5236417	187	239	359	422	781	0.4597	(0.4723, 0.5728)	Hap 1
			5236740	145	175	145	175	320	0.4531	(0.3999, 0.6452)	Unclassified
			5237284	89	76	234	251	485	0.4825	(0.4417, 0.6035)	Unclassified
			5243757	123	132	357	383	740	0.4824	(0.4695, 0.5756)	Unclassified
			5244144	47	56	404	439	843	0.4792	(0.4760, 0.5691)	Unclassified
			5244299	28	39	432	478	910	0.4747	(0.4794, 0.5657)	Hap 1
			5245406	132	165	132	165	297	0.4444	(0.3904, 0.6547)	Unclassified

			5245507	114	145	246	310	556	0.4424	(0.4520, 0.5931)	Hap 1
			5246000	189	189	189	189	378	0.5000	(0.4187, 0.6264)	Unclassified
			5246042	205	187	394	376	770	0.5117	(0.4716, 0.5735)	Unclassified
			5246203	226	225	620	601	1221	0.5078	(0.4904, 0.5547)	Unclassified
			5246512	168	180	788	781	1569	0.5022	(0.4975, 0.5476)	Unclassified
			5247141	126	127	914	908	1822	0.5016	(0.5010, 0.5441)	Unclassified
			5247733	138	182	1052	1090	2142	0.4911	(0.5042, 0.5409)	Hap 1
			5247791	194	208	194	208	402	0.4826	(0.4249, 0.6202)	Unclassified
			5248243	211	186	405	394	799	0.5069	(0.4734, 0.5717)	Unclassified
			5248641	188	182	593	576	1169	0.5073	(0.4890, 0.5561)	Unclassified
			5248770	140	116	733	692	1425	0.5144	(0.4950, 0.5501)	Unclassified
			5248842	30	59	763	751	1514	0.5040	(0.4966, 0.5485)	Unclassified
			5257189	160	156	923	907	1830	0.5044	(0.5011, 0.5440)	Unclassified
			5259000	243	140	1166	1047	2213	0.5269	(0.5048, 0.5403)	Unclassified
F13	Father	type α SNPs	5225021	129	0	129	0	129	1.0000	(0.9728, 0.9998)	Hap 0
			5228143	618	0	618	0	618	1.0000	(0.9835, 0.9891)	Hap 0
			5228708	628	0	628	0	628	1.0000	(0.9835, 0.9891)	Hap 0
			5229196	589	0	589	0	589	1.0000	(0.9834, 0.9893)	Hap 0
			5236792	559	0	559	0	559	1.0000	(0.9832, 0.9894)	Hap 0
			5236851	612	0	612	0	612	1.0000	(0.9835, 0.9892)	Hap 0
			5253586	571	0	571	0	571	1.0000	(0.9833, 0.9894)	Hap 0
	Mother	type	5227950	289	236	289	236	525	0.5505	(0.4665,	Unclassified

		α SNPs								0.5887)	
			5227975	257	234	546	470	1016	0.5374	(0.4960, 0.5591)	Unclassified
			5229743	187	220	733	690	1423	0.5151	(0.5050, 0.5501)	Unclassified
			5231897	364	296	1097	986	2083	0.5266	(0.5122, 0.5430)	Unclassified
			5232573	337	248	1434	1234	2668	0.5375	(0.5156, 0.5396)	Unclassified
			5236417	294	283	1728	1517	3245	0.5325	(0.5177, 0.5375)	Unclassified
			5247876	301	244	2029	1761	3790	0.5354	(0.5191, 0.5360)	Unclassified
			5249004	260	172	2289	1933	4222	0.5422	(0.5200, 0.5352)	Hap 0
		type β SNPs	5249290	256	258	256	258	514	0.4981	(0.4100, 0.5348)	Unclassified
